# A *Saccharomyces cerevisiae* knockout screen for genes critical for growth under sulfur- and nitrogen-limited conditions reveals intracellular sorting via vesicular transport systems

**DOI:** 10.1093/g3journal/jkaf074

**Published:** 2025-04-10

**Authors:** Sean Simmons, Joseph Graham, David Cobb, Shreya Sudakar, Emma G Teng, Emily Lee, Elaine T Do, Logan Scott, Allison Price, Perry Kezh, Amy M Wiles

**Affiliations:** Department of Biology, Mercer University, Macon, GA 31207, USA; Department of Biology, Mercer University, Macon, GA 31207, USA; Department of Biology, Mercer University, Macon, GA 31207, USA; Department of Biology, Mercer University, Macon, GA 31207, USA; Department of Biology, Mercer University, Macon, GA 31207, USA; Department of Biology, Mercer University, Macon, GA 31207, USA; Department of Biology, Mercer University, Macon, GA 31207, USA; Department of Biology, Mercer University, Macon, GA 31207, USA; Department of Genetics, University of Georgia, Athens, GA 30602, USA; Department of Biology, Mercer University, Macon, GA 31207, USA; Department of Biology, Mercer University, Macon, GA 31207, USA; Department of Biomedical Sciences and Pathobiology, Virginia Tech, Blacksburg, VA 24061, USA; Department of Biology, Mercer University, Macon, GA 31207, USA

**Keywords:** *Saccharomyces cerevisiae*, limited nutrients, nitrogen, sulfur, knockout screen, pathway analysis, vesicular transport, intracellular sorting, phosphatidylinositol, endosome, autophagy

## Abstract

It is understood that nutrient availability significantly impacts cellular growth and metabolism. The genetic basis for survival in nutrient-limited conditions, however, is not as thoroughly explored. The identification and description of the genes vital for growth in these conditions would therefore enhance the understanding of the signaling and biochemical pathways and processes crucial for cellular survival and growth under these constraints. A growth screen of a gene deletion library representing 4,934 genes of *Saccharomyces cerevisiae* was completed to discover genes required for normal growth under sulfur- and nitrogen-limited conditions. Genes were identified as required under these restrictive environments based on a comparison with their growth in a synthetic, defined control medium. After normalization and statistical analysis, 732 genes were noted as essential in sulfur-limited medium, and 761 genes were found for nitrogen-limited medium, with an overlap of 313 genes found to be needed in both, significantly more than expected by chance. Kyoto Encyclopedia of Genes and Genomes and Gene Ontologies were analyzed to investigate those processes involved. Proteins identified act in central metabolism and in metabolism of amino acids, glycerolipids, glycerophospholipids, and vitamins and in the pathways of mitogen-activated protein kinase and phosphatidylinositol signaling and the processes of vesicle trafficking, autophagy, mitophagy, and endocytosis. Of these, the metabolism and signaling of phosphatidylinositols are not frequently identified in screens examining nutrient starvation in yeast, nor are vesicular fusion, endocytosis, or trafficking to the early endosome, as we have discovered here. This study invites further exploration into the roles of these processes in adaptation to nutrient stress.

## Introduction

The effects nutrients have on cellular growth and the metabolic and signaling pathways involved are a compelling, albeit challenging, area of study due to the interwoven nature of biological pathways (Amtmann and Armengaud 2009). A wide array of nutritious laboratory media for the growth of *S. cerevisiae* have been developed, ranging from minimal synthetic to rich—a common requirement for all being biologically available nitrogen, sulfur, and carbon sources, among other trace elements. When grown in nutrient-deprived conditions, organisms are forced to adapt, often resulting in changes to gene expression levels. The effect of stress and starvation conditions on gene expression in *Saccharomyces cerevisiae* has been widely studied, and comprehensive reviews highlight emerging trends that tie gene expression to environmental conditions (Gasch and Werner-Washburne 2002; Taymaz-Nikerel *et al*. 2016). In *S. cerevisiae*, it has been well observed that nutrient starvation affects transcription levels, with the carbon source having the most dramatic impact on expression pattern (Wu *et al*. 2004). Along with lower carbon availability, nitrogen deprivation impacts regulation responses, affecting translation and leading to cell cycle arrest (Rødkaer and Faergeman 2014). Few studies have limited sulfur, and those that explored its role have primarily focused on its metabolism (Ohtsuka *et al*. 2021) or restricted the study to gene expression (Boer *et al*. 2003). Those that have investigated the effect of gene deletion in combination with limited nitrogen availability have focused on steady state growth (Taymaz-Nikerel *et al*. 2016) or on fermentation as related to wine production (Peter *et al*. 2018). Although studies have provided a solid understanding of the transcriptional response to carbon, nitrogen, and amino acid starvation, they do not address which genes are essential for culture growth under sulfur or nitrogen starvation conditions, and these genes have not been identified.

Identifying genes that allow the microorganism to respond effectively to an environment deficient in nutrients by supporting growth would allow insight into the pathways essential for growth when these resources are not in abundance. Therefore, we ask in this study what genes are required for culture growth under nutrient-limited conditions rather than what effect the conditions have on gene transcription by screening a knockout library of 4,934 genes under limited nutrients conditions when compared with culture growth in defined medium. Under these limiting conditions, normal growth can be observed for wild-type cells for at least a short period (Wiles *et al*. 2006). This knockout screen revealed numerous *S. cerevisiae* genes essential to support growth under sulfur- or nitrogen-limited conditions whose further study may be warranted. These genes were then mapped to pathways in which their encoded proteins are found (Kanehisa *et al*. 2023) and to Gene Ontologies (GOs) in order to understand what role the genes and their products might have in promoting survival under these nutrient-limited conditions. Our investigation leads us to a rudimentary understanding of the significance of metabolic and signaling pathways required during starvation conditions.

## Materials and Methods

### Screen for growth differences in nutrient-limited media

In a 96-well plate format and after 48 h of growth upon being removed from −80°C storage, 9 copies of a haploid *Saccharomyces* genome deletion library (Invitrogen, Carlsbad, CA, USA) in the strain backgrounds BY4742 (*MAT*α *his3*Δ*1 leu2*Δ*0 lys2*Δ*0 ura3*Δ*0*) and BY4739 (*MAT*α *leu2*Δ*0 lys2*Δ*0 ura3*Δ*0*) were pin-replicated from a YEPD (1% yeast extract, 2% peptone, and 2% dextrose) culture, which had been washed with water, into 200 μL synthetic defined (SD) medium [being yeast nitrogen base (YNB) without amino acids (BD Difco, Franklin Lakes, NJ, USA) + 2% glucose + HLKU (20 μg/mL histidine, 30 μg/mL leucine, 30 μg/mL lysine, and 20 μg/mL uracil)] with a 4-μL inoculum and grown for 18 h at 30°C while shaking, such that the majority of the strains had likely entered log phase. Medium was removed via centrifugation at 3,000 rpm at 4°C for 5 min followed by vacuum with a 12-channel manifold (V&P Scientific, Inc., San Diego, CA, USA) at the top edge of the wells. Two-hundred microliters of new media were added to each plate such that the screen was performed in triplicate: SD, SD with limited sulfur (“limS,” SD with all sulfur-containing salts replaced with corresponding chloride salts), and SD with limited nitrogen (“limN,” SD with ammonium sulfate replaced with sodium sulfate), and the contents were stirred to resuspend. These experimental media were assembled following Wickerham's formulations (Wickerham and Burton 1948; Wickerham 1951) on the same day as the assay and modified to limit nutrients as previously described, such that limS did not contain sulfur and limN only had nitrogen contained within essential vitamins and amino acids and therefore nonaccessible (Wiles *et al*. 2006). At time 0, the pH of all media fell between 4.83 and 4.96. After 4 h of growth at 30°C while shaking, cell OD was read at 600 nm using an Epoch spectrophotometer (BioTek, Winooski, VT, USA). All bench work was performed manually.

### Data analysis

Data were normalized by background subtraction (of empty wells) followed by quantile normalization (BSQN) and by using the R package cellHTS2, both as previously described (Boutros *et al*. 2006; Wiles *et al*. 2008). Comparisons made between limited nutrient and control media were evaluated such that those that grew worse in the limited medium resulted in negative values (*M*-values were calculated from BSQN, and difference was calculated from cellHTS2). A moderated *t*-test (R x64 4.0.2, BiocManager library, limma package, Ritchie *et al*. 2015) was used to identify genes removed from any deletant with a *P*-value of <0.05 and a negative calculated coefficient of variation for limS, limN, or both under either of the data normalization methods. Although normalization and statistics were conducted on all wells of each library plate, in cases of genes represented within the library more than once, we relied upon data from the revised knockout strains present in the 2.0 library plates since their redevelopment sought to overcome inaccuracies in the original version of the library (Giaever and Nislow 2014). The *Saccharomyces* Genome Database (SGD) was relied upon to obtain gene names from provided systematic names and to identify paralogs (Wong *et al*. 2023). The Kyoto Encyclopedia of Genes and Genomes KEGG Mapper (Kanehisa *et al*. 2022) was used to retrieve pathways in which proteins encoded by identified genes were found as represented by their systematic name (KEGG release 110.0), and KEGG Brite (Kanehisa *et al*. 2023) was used to recover classifications of proteins not included in KEGG pathways. The PANTHER Overrepresentation Test (Ashburner *et al*. 2000; Mi *et al*. 2019; Thomas *et al*. 2022; The Gene Ontology Consortium *et al*. 2023) was used to retrieve GOs from PANTHER GO-Slim of all tested and identified genes as represented by their SGD ID (PANTHER release 19.0). GO-Slim overrepresentation tests were also conducted at PANTHER using Fisher's exact test with a false discovery rate correction.

### Verification

Following identification of deletants, 184 identified as required for growth in limS, limN, or both were chosen for verification in duplicate, as described above, at 30 and 37°C. The strain BY4700 (MAT**a**  *ura3*Δ*0*) was also included as a control, and optical density (OD) was measured hourly for 14 h. Data were normalized by determining the average OD of all wells for each strain at time 0 and multiplying all further readings of each well by the factor necessary to set it to the strain's average at time 0. In this manner, variations between starting concentrations between replicates and conditions were taken into account. Seven of these strains were randomly chosen for further verification and were grown at 30°C while shaking at 220 rpm for 12 h in 50 mL of each of the 3 media. Experimental media were inoculated at 5 × 10^5^ cells/mL from water stocks made from cultures grown overnight in SD, washed once, and held at 4°C for no more than 2 days. Every 4 h during the assay, triplicate aliquots were taken, with 2 serial diluted for cell counting via hemocytometer, and the third serial was diluted followed by plating 5 μL spots onto YEPD agar. Plates were grown for 30 h at 30°C.

## Results and discussion

### Identification of genes required for growth in limited nutrient media

The *S. cerevisiae* genome deletion libraries have commonly been used to investigate the requirement of genes under various environmental conditions (Giaever and Nislow 2014). Here, we have conducted a screen on a gene deletion library of 4,934 *S. cerevisiae* strains to identify those genes required for growth in defined media, either sulfur-free or nitrogen-limited media (limS and limN, respectively) in order to investigate genes and their products novel to responding to limited nutrients. We relied upon the *MAT*α haploid library because the *MAT***a** library was auxotrophic for the sulfur-containing amino acid methionine. A screening protocol was therefore developed to test this library.

The time of 4 h was chosen because the authors previously found that wild-type cells show no difference in growth in these media until either at 6 hr (limN) or after 8 h (limS, with the beginnings of decline at 8) (Wiles *et al*. 2006). We wanted to capture the difference while cultures were still in log phase but before there was a considerable difference in wild-type growth between starvation and control media; 4 h was chosen as an intermediate point.

The process to remove overnight SD medium began with centrifugation. After this initial centrifugation, 0, 1, and 2 washes with sterile water were tested, and cell loss was calculated for each. No statistical difference in the percentage of cell growth in limS or limN was observed, however, suggesting that a sufficient amount of overnight medium was able to be removed after the initial centrifugation so as not to affect subsequent growth, and the decision was made to forgo washing.

### Genes required for growth in limited sulfur and limited nitrogen show considerable overlap

Data were normalized by 2 methods: cellHTS2 (Boutros *et al*. 2006) and BSQN (Wiles *et al*. 2008; Ritchie *et al*. 2015). Both use different metrics and detect different aspects of the data. cellHTS2 involves a plate median normalization, while BSQN begins with subtracting the values of wells without a strain on the plate from each well containing a strain, followed by quantile normalization, which sorts a dataset to give replicates the same statistical properties. In screens examining cell culture growth, less growth than that found in control conditions is frequently expected rather than a normal distribution; cellHTS therefore allows for outliers, while quantile normalization expects a normal distribution since it was developed for gene expression analysis. We have shown that taken together, these 2 normalization methods provide the most robust set of true positives (Wiles *et al*. 2008). To account for variations in cell size or growth rates between different deletion strains that may have affected the OD of cultures, ODs of each strain were only compared with ODs of the same strain, and direct comparisons between strains were not carried out. In this way, any differences in OD measurements due to strain variation were avoided. Upon comparing the normalized growth of deletants using a moderated *t*-test, we identified genes from those deletants whose growth in limited nutrient conditions was significantly lower than in control medium (*P* < 0.05 with a negative coefficient), finding 732 to affect growth in limS and 761 affecting growth in limN (Supplementary Tables 1 and 2).

If a deletant showed the phenotype of statistically less growth in one of the nutrient-limited media when compared with its growth in control conditions after either BSQN or cellHTS2 normalization, the deleted gene was determined to be vital for that environment. Of these, the deletion of 313 genes was found to affect growth under both limiting conditions. With 4,934 genes tested and the fractions of those found by each screen (0.148 and 0.154), a fraction of only 0.0229, or 113 genes, was expected to be found in both. A chi-square test with 3 degrees of freedom (from the 4 categories of genes found to affect cell concentration in both screens, genes found only in limS, genes found only in limN, or genes found in neither) was therefore conducted, yielding a *P*-value of <0.0001 demonstrating a statistically different overlap than expected by chance. At least part of the 313 overlapping genes may, then, contribute to a more general nutrient starvation response.

**Fig. 1.**
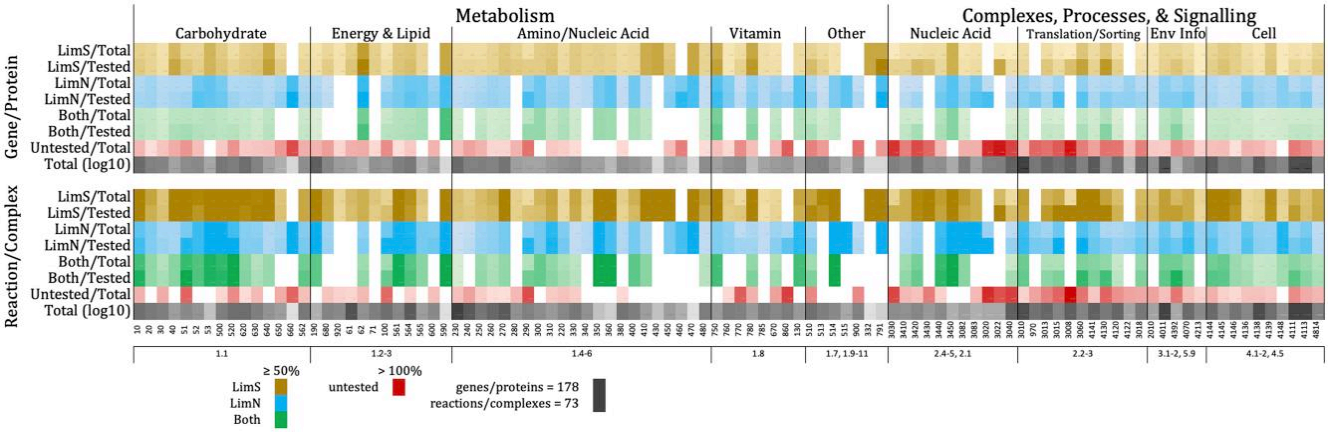
Percentage of KEGG pathways affected by growth in limS and limN. All KEGG pathways in which at least one deletant was found to have statistically less growth in at least one limiting medium when compared with growth in SD are represented and ordered in columns as indicated by their KEGG identifier and KEGG classification (1 metabolisms: 1.1 carbohydrate, 1.2 energy, 1.3 lipid, 1.4 nucleotide, 1.5 amino acid, 1.6 other amino acid, 1.8 cofactor and vitamin, 1.7 glycan, 1.9 terpenoids and polyketides, 1.10 other secondary metabolites, 1.11 xeobiotic; 2 genetic information processing: 2.4 replication, repair, 2.5 chromosome, 2.1 transcription, 2.2 translation, 2.3 folding, sorting, degradation; 3 environmental information processing: 3.1 membrane transport, 3.2 signal transduction; 5 organismal systems: 5.9 aging; 4 cellular processes: 4.1 transport, catabolism, 4.2 cell growth, death, 4.5 cell motility). Tints of colors represent the percentage of the genes/proteins found to be statistically relevant in limS (yellow), limN (blue), or both (green) in either the total *S. cerevisiae* genes/proteins annotated at being in the KEGG pathway or in those that were annotated and tested (nonessential). Since several proteins are occasionally annotated as having the ability to carry out the same reaction or as being part of a protein complex, the same is shown for reactions and complexes rather than single genes/proteins. The darkest color represents at least 50% coverage of the pathway. Also indicated is the percentage of the pathways that were untested because of essential genes or because all proteins in a complex or carrying out a reaction were essential (red). Finally, a representation of the total number of genes/proteins or reactions/complexes annotated in a given KEGG pathway is given log in grayscale, with the maximum number in black.

KEGG metabolic and signaling pathways (Kanehisa 1997) were then queried for overrepresentation of these 1,180 identified genes through KEGG Mapper (Kanehisa *et al*. 2022). Unsurprisingly, not all genes identified in these 2 screens are found in KEGG pathways. Indeed, only 337 of our identified gene products are annotated as a member of a KEGG pathway, with 194 limS gene products, 231 limN products, and 88 found in both screens. KEGG pathways with a substantial portion of their components found in at least one of our screens are discussed below, organized by the general category of metabolic pathway or by type of complex, process, or signaling pathway to group together proteins that may directly or indirectly interact (Fig. 1; Supplementary Tables 2 and 3). Here, we explore possibilities as to why, when knocked out, each gene results in decreased culture growth when starved for sulfur, nitrogen, or both when compared with the growth of its strain in SD medium. For cellular function, both metabolic and signaling pathways interact through cross-talk, and enzymes may have more than one function, resulting in some proteins noted as being in more than one pathway. The locations of the multipathway proteins are noted throughout, be it adjacent to other identified proteins or connecting their pathway to an adjacent pathway. Numbers given in parentheses are those of the KEGG pathway identifier, and systematic names and identifying screens are given for genes/proteins whose deletant showed statistically reduced cell concentration compared with its growth in SD medium. Proteins discussed but not identified in one of our screens are noted as “not found,” while those that we discuss but were not available for testing because they were essential and therefore not present in the deletion library are marked as “not tested.”

In addition, we queried our dataset for genes encoding transcription factors, which are unlikely to be annotated in KEGG, using the 190 transcription factor genes (Gordân *et al*. 2011) curated from other sources. Genes encoding 168 of these proteins were represented in the library, and all were annotated as being verified or uncharacterized. Between our screens, we identified 39, with 27 by limS, 23 by limN, and 11 were found in both screens.

Most genes we found are annotated as “verified” by SGD, while some are still confirmed to be genes but are as of yet “uncharacterized.” There are, however, 90 that are annotated as “dubious” or carry another annotation suggesting that they are unlikely to be transcribed (Fisk *et al*. 2006). We have chosen to retain these genes in our dataset for future researchers who may find them of benefit.

### Metabolisms

We have grouped the KEGG metabolic pathways discussed below into metabolisms of amino acids and nucleotides, carbohydrates, lipids, multimacromolecules (those compounds with more than one type of macromolecule), and vitamins (Fig. 1; Supplementary Table 3). Below we indicate numbers of genes whose proteins act in a pathway, but we also discuss the number of reactions. Enzymes may catalyze more than one reaction, and some reactions may be catalyzed by more than one peptide or protein.

### Role of amino acid metabolism

Quite a number of amino acid metabolic pathways were found to contain proteins required for culture growth under limited sulfur and/or nitrogen conditions—arginine and proline (220 and 330); alanine, aspartate, and glutamate (250); glycine, serine, and threonine (260); cysteine and methionine (270); valine, leucine, and isoleucine (280 and 290); lysine (300 and 310); histidine (340); and tyrosine, phenylalanine, and tryptophan (350, 360, 380, and 400). We expected to find some proteins carrying out amino acid catabolism reactions to be of consequence since eliminating ammonium from the medium would likely cause cells to rely upon nitrogen contained within amino acids and their metabolites, but our screens also revealed the necessity of some anabolic reactions as well. With the addition of lysine, leucine, and histidine in abundance, we did not expect many genes to be of importance in the related pathways. The auxotrophies of the strains used and the addition of the amino acids, however, may have had further effects on growth in the absence of genes encoding proteins in these pathways. Of the amino acid metabolic pathways revealed, cysteine metabolism was particularly notable.

Of interest, one gene encodes a protein with activity in 7 metabolic pathways listed above (220, 250, 270, 330, 350, 360, and 400)—*AAT1* (YKL106W, limS and limN) encodes the mitochondrial aspartate aminotransferase Aat1p, which converts between oxaloacetate and aspartate and conducts similar transferase reactions and sits at important positions throughout these amino acid synthesis pathways (Lee *et al*. 2013). While the orthologous protein in human endothelial cells, GOT1/AAT1 (the orthologous protein in human endothelial cells), is a transaminase, it has also been shown to produce sulfur dioxide, which then has an inhibitory effect on the enzyme (Song *et al*. 2020). Here, yeast *AAT1* was found to be required when grown in both limS and limN, potentially making it a linchpin in amino acid synthesis during starvation conditions. Aat1p is a component of the malate-aspartate NADH shuttle, which also includes mitochondrial Mdh1p (YKL085W, limS). Malate dehydrogenases are further discussed with the citrate cycle (20) below, but with both peptides being involved in amino acid metabolic pathways, this shuttle complex ties amino acid production to central metabolism through oxaloacetate and the movement of nicotinamide adenine dinucleotide (NAD/NADH).

Three other genes encode proteins found in a multitude of amino acid metabolic paths: *ALD3* (YMR169C, limS), *ALD4* (YOR374W, limS), and *ARO8* (YGL202W, limN). These 3, however, were only required for growth in 1 of the 2 limiting conditions. Cytoplasmic Ald3p and mitochondrial Ald4p are aldehyde dehydrogenases (ALDs), which work in 6 pathways (280, 310, 330, 340, 380, and 410) to convert aldehydes to their carboxylic acids, an example of which is their conversion between acetate and acetaldehyde (Boubekeur *et al*. 2001). Both the cytoplasmic Ald3p and its paralog Ald1p (not found) are also involved in pantothenic acid synthesis (410 and 770, below); however, despite sharing most functionalities, evidence supports Ald3p being more important than Ald2p in this synthesis (White *et al*. 2003). The mitochondrial Ald4p is the major mALD in yeast, responsible for 80% of the functional enzymatic activity, while Ald5p (not found) provides the remaining 20% (Misonou *et al*. 2014). These ALDs are also found in glycerolipid (561), ascorbate and aldarate (53), and pyruvate (620) metabolic pathways, as discussed below. *ARO8* encodes a transaminase involved in another 6 amino acid pathways (270, 300, 350, 360, 380, and 400) and ubiquinone biosynthesis (130). It is an aromatic aminotransferase in *S. cerevisiae* whose products include phenylalanine, tyrosine, tryptophan, and methionine (Deed *et al*. 2019).

In addition to *AAT1* and *ARO8* in the KEGG pathway cysteine and methionine metabolism (270), several other genes were of statistical relevance for 11 in all, accounting for 27% of the pathway's proteins that were tested. Aside from Aro8p, only 3 gene products involved in methionine biosynthesis were found here. Mde1p (YJR024C, limS) is a 5′-methylthioribulose-1-phosphate dehydratase, which is involved in the methionine salvage pathway (abbreviated as the MTA cycle for 5′-methylthioadenosine). The MTA pathway is used by eukaryotic cells to regenerate methionine by using a byproduct from S-adenosylmethionine reactions (Pirkov *et al*. 2008). Adi1p (YMR009W, limS) is a yeast aci-reductone dioxygenase homolog, which, in other eukaryotic organisms, converts aci-reductone into metabolites used in the MTA cycle (Hirano *et al*. 2005). KEGG lists both Mde1p and Adi1p as functioning exclusively in this pathway, intimating that the MTA cycle is therefore required in response to sulfur starvation. Aro8p (limN) is also possibly involved in the MTA pathway as a MOB transaminase (Pirkov *et al*. 2008).

**Fig. 2.**
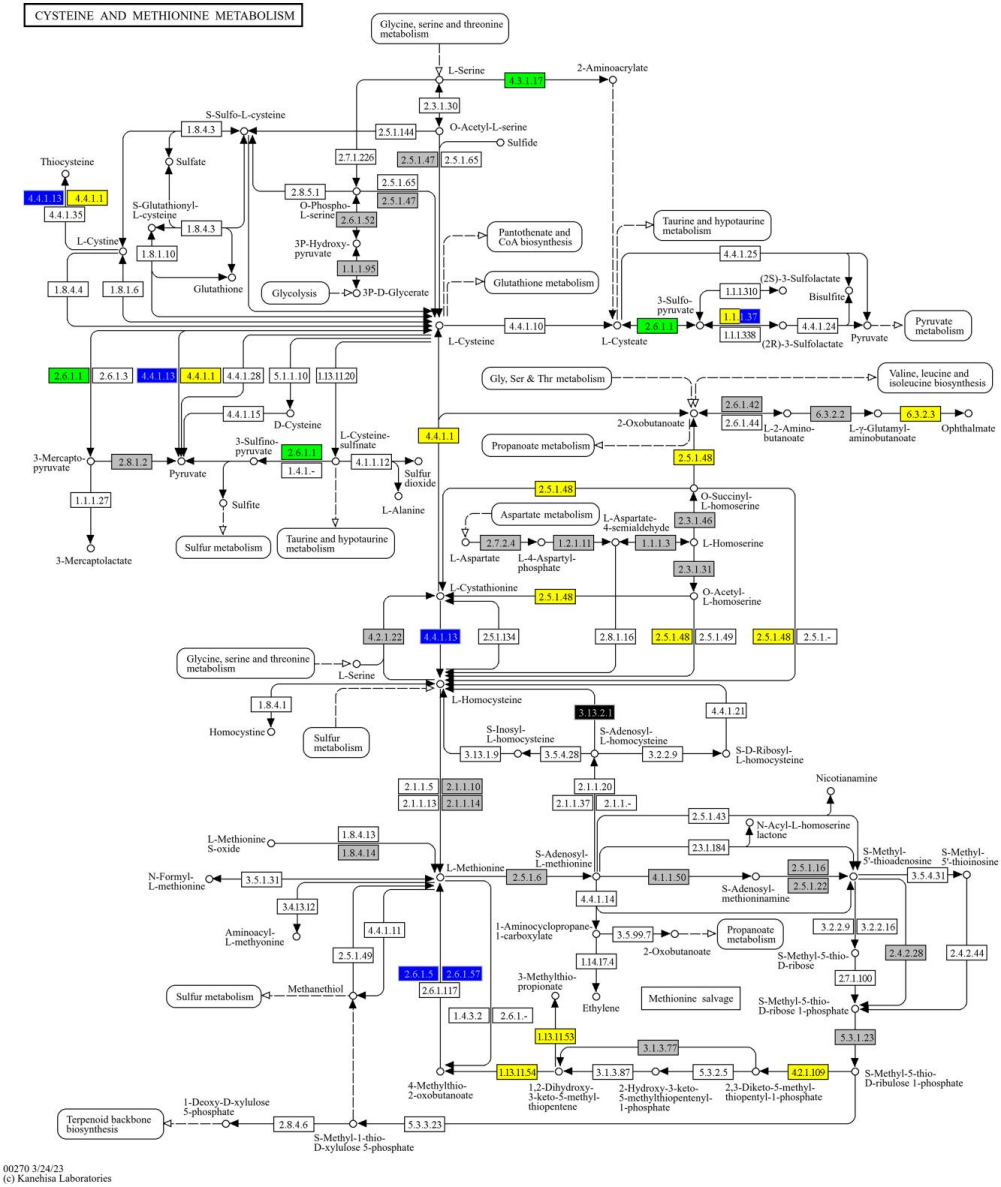
KEGG pathway sce00270 cysteine and methionine metabolism. Colors represent at least one protein annotated at the node/reaction had statistical relevance in limS only (yellow), in limN only (blue), or in both limS and limN (green). Those nodes that were tested but no proteins were found to be relevant are gray, and those nodes present in *S. cerevisiae* but were untested are black. Nodes in white are not annotated in KEGG to exist in *S. cerevisiae*. Image © KEGG (Kanehisa *et al.* 2022).

The cysteine side of the KEGG pathway is implicated more strongly in survival in our screens than is the methionine side. The cysteine and methionine reactions converge at homocysteine. Of the 29 testable reactions on the cysteine side of homocysteine, we identified 15 as involving proteins required for normal growth when cells were starved for nutrients as opposed to finding only 4 of the 12 methionine-related reactions (Fig. 2). *YML082W* (limS) is known to encode a protein important for n-propanol metabolism and synthesis (Wang *et al*. 2021). Cha1p (YCL064C, limS and limN), catabolic serine/threonine deaminase, functions to maintain homeostasis in the cell by maintaining serine levels. When there is an overabundance of serine in the cell, Cha1p positively regulates sphingolipid synthesis (Zuzuarregui *et al*. 2006), thereby decreasing the amount of free serine available. *CHA1* expression is positively regulated when grown on serine as a sole nitrogen source (Petersen *et al*. 1988). Since we did not identify the deaminase specific for threonine (Ilv1p) as significant, it appears that the use of serine is of importance here. Malate dehydrogenases, both the mitochondrial Mdh1p (YKL085W, limS) and the peroxisomal Mdh3p (YDL078C, limN) versions, play different roles in maintaining homeostasis: in the reduction of oxaloacetate during NADH oxidation and during peroxisomal redox reactions, respectively (Kurita 2003). The cytoplasmic variant, Mdh2p, was not found in these screens. These enzymes are also found in carbon metabolic pathways discussed later (20, 620, and 630).

Glutathione synthetase (Gsh2p, YOL049W, limS) functions in cysteine metabolism to anabolize ophthalmate, a compound similar to glutathione (GSH). Stepping away from cysteine metabolism briefly, we note that Gsh2p also acts in glutathione metabolism (480) carrying out the second step of the GSH synthesis pathway to generate GSH (Xu *et al*. 2017). Glutathione is then involved in a variety of cellular pathways, including antioxidation, detoxification, and amino acid transport. Glutathione peroxidase (Ure2p, YNL229C, limS and limN), the first step in one catabolic pathway breaking GSH into its constituent amino acids (Bai *et al*. 2004), was also significant in our screens. This underscores the importance of glutathione metabolism when sulfur is limited. The peroxidase in particular is likely to function here as a means by which to access cysteine in the absence of free, biologically available sulfur and perhaps all 3 constituent amino acids when nitrogen is limited. Although KEGG lists the peptide Rnr4p (YGR180C, limS) as part of a complex that oxidizes trypanothione, a compound composed of 2 GSH molecules linked by spermidine, which acts in place of glutathione in trypanosomatid species, there is evidence that *S*. *cerevisiae* does not make this compound (Oza *et al*. 2002), and the complex likely only functions in purine and pyrimidine metabolism (230 and 240), discussed later.

The last 3 cysteine and methionine metabolism proteins whose genes were found to be required for growth in a limited medium were Irc7p (YFR055W, limN), Cys3p (YAL012W, limS), and Yml082 (limS). Irc7p is an aminotransferase that enables yeast to use cysteine, but not methionine, as a nitrogen source for growth (Santiago and Gardner 2015), making its importance in limN survival clear. It catalyzes the conversion of cystathionine to homocysteine. Cystathionine γ-lyase is part of the reverse trans-sulfuration pathway in yeast, with its gene, *CYS3*, transcribed in yeast when the cell is under sulfur starvation conditions and is repressed when the cells contain high amounts of cysteine (Hiraishi *et al*. 2008). *YML082W* is predicted to encode a cystathionine γ-synthase involved in the anabolism of cystathionine compounds from cysteine compounds (Wang *et al*. 2021). All 3 of these proteins are also involved in selenocompound metabolism (450), not discussed here. We found only one other required gene whose product functions in selenocompound metabolism: *MSM1* (YGR171C, limN) encodes a mitochondrial methionyl tRNA-synthetase. It has been found, however, that *MSM1* is nonessential, and its deletion causes respiratory deficiency but not cell vitality deficiency under normal conditions (Tzagoloff *et al*. 1989). Here, the stress of growth in limited nitrogen may be too much for the cell to grow well without it.

The KEGG pathway β-alanine metabolism (410) also proved interesting in that the proteins found in our screen were clustered within it, and all 4 were found to be required for culture growth when starved for sulfur. In addition to *ALD3* and *ALD4*, the ALDs discussed above, *FOX2* (YKR009C, limS and limN) and *GAD1* (YMR250W, limS) were found to be required in limited media. Fox2p has both 3-hydroxyacyl-CoA dehydrogenase and enoyl-CoA hydratase activity for its role in β-oxidation in the peroxisome and is required for support of normal growth in oleic acid medium (Gabriel *et al*. 2014). Although we show its importance for growth in media absent of sulfur and limited nitrogen, further experimentation with other media would be beneficial to understanding the physiological role of this gene. *GAD1* encodes a putative glutamic acid decarboxylase. Specifically, these enzymes catalyze the production of γ-aminobutyric acid from glutamic acid, but others with the same Enzyme Commission (EC) number (4.1.1.15) convert aspartate into β-alanine. Mutations in *GAD1* can cause pyridoxine deficiency, and *gad1Δ* extends the lifespan of yeast under normal growth conditions (Kamei *et al*. 2011). It is important to note that our assay did not measure life span, measured in budding yeast by the number of cell divisions a mother cell undergoes, but rather changes in cell concentration over 4 h.

In arginine biosynthesis (220), 2 additional genes were of interest. *ALT1* (limS and limN) encodes a vital alanine transaminase whose deletion has been found to reduce the cell's lifespan (Yu *et al*. 2013). *DUR12* (YBR208C, limN) encodes a protein with several homologs acting as biotin carboxylases or acetyl-CoA carboxylases and other biotin-dependent carboxylases (Kanamori *et al*. 2004). Dur12p is responsible for degrading urea to ammonium. KEGG's atrazine degradation pathway (791) is where one finds the catabolism of cyanamide into urea and the anabolism of urea into allophanate and to carbon dioxide. Cyanamide is acted upon by Ddi2p (YNL335W, limS) and Ddi3p (not tested), both cyanamide hydratases (Li *et al*. 2019). Also functioning in this pathway is Dur12p. Here, Dur12p acts as an allophanate carboxylase. Although we identified these 2 genes as important under different limiting conditions, they were the only 2 genes in this pathway in yeast that were testable, since *DDI3* was absent from the deletion library. Therefore, we found all testable yeast genes involved in this KEGG pathway to be required for growth under a starvation condition.

Lys21p (YDL131W, limS and limN) and Lys20p (not found) are isoforms of a homocitrate synthase that initiates the first step in the lysine biosynthesis pathway (300 and 310) from oxoglutarate from the citrate cycle. Though the 2 isoforms have redundant functionality, Lys21p is more efficient at initiating the synthetic pathway than Lys20p when cells are grown on ethanol (Quezada *et al*. 2008). Lys9p (YNR050C, limS and limN), the enzyme at the penultimate step of lysine synthesis, was also found, along with Aro8p. Finally, Ctm1p (YHR109W, limN) is an N-methyltransferase that tri-methylates lysine residues of Cyc1p (YJR048W, limS) (Polevoda *et al*. 2000), discussed in oxidative phosphorylation (190) below. In other species, this reaction can lead to reclamation of the lysine, but yeast do not appear to have the necessary enzymes to continue the process. However, little is known about the function of Ctm1p. It is interesting that 3 of the 10 steps of the lysine biosynthetic pathway were determined to be important in our nitrogen-limiting screen, 2 of which were also found when deletants were challenged to grow in the absence of sulfur, particularly because lysine was provided in abundance due to the strains’ *lys2* auxotrophy. This pathway does not branch, and the first reaction, carried out by Lys21p, appears to be directional, suggesting a dependence upon other metabolites in the pathway, such as homocitrate, the product of Lys21p.

### Role of nucleotide metabolism

Components of both purine and pyrimidine metabolism (230 and 240, respectively) were discovered to be important not only when cells were starved for nitrogen but also starved for sulfur. While some gene products were deemed crucial to survival in limN, most found were identified due to their reduced growth in limS. Rnr4p (YGR180C, limS) is a subunit of ribonucleotide-diphosphate reductase and is involved in both pathways, converting guanosine diphosphate (GDP), adenosine diphosphate (ADP), cytidine diphosphate (CDP), and uridine diphosphate (UDP) to their 2′-deoxy-bases, respectively, thereby being responsible for the generation of deoxynucleotide triphosphates (dNTPs). Rnr4p forms a homodimer to bind the ribonucleotide reductase's cofactor (Sommerhalter *et al*. 2004). It also participates in glutathione metabolism (480), discussed above. Its paralog, Rnr2p, was not tested.

Specific to purine metabolism, the high-affinity cyclic adenosine monophosphate (cAMP) phosphodiesterase Pde2p (YOR360C, limS) converts 3′,5′-cGMP (cyclic guanosine monophosphate) to guanosine monophosphate (GMP). The low-affinity enzyme, Pde1p, was not so crucial to be found in our screens, but the high-affinity Pde2p was, and it is a controlling factor in basal levels of cAMP (Ma *et al*. 1999). The enzyme that would carry out the similar reaction for cytidine monophosphate (CMP) does not seem to exist in *S. cerevisiae*. While present in purine metabolism, the oxidoreductase Ora1p (YMR226C, limS) also acts in glycine and threonine metabolism (260), generating an immediate precursor to each one of these amino acids and 3-hydroxypropanoate (Fujisawa *et al*. 2003). Additionally, 2 enzymes involved in purine salvage that produce xanthosine monophosphate (XMP) were identified in our screens. *IMD2* (not tested), *IMD3* (YLR432W, limS), and *IMD4* (YML056C, limS and limN) are paralogs that each encode an inosine monophosphate dehydrogenase, which acts as the rate-limiting step in guanosine triphosphate (GTP) production, converting inosine monophosphate (IMP) to XMP (Hyle *et al*. 2003). Although neither *IMD3* nor *IMD4* are required for growth under standard laboratory conditions, limiting nutrients here requires the functioning of at least one of these genes. *HAM1* (YJR069C, limS) encodes the second enzyme that we found statistically relevant that produces XMP from xanthosine triphosphate (XTP); Ham1p is also involved in the conversion of the inosine monophosphates ITP/dITP to IMP/dIMP (Stepchenkova *et al*. 2009). From XMP, GMP is synthesized by Gua1p (not tested). In addition, the production of phosphoribosyl pyrophosphate (PRPP) also seems of import in that genes encoding components of 2 enzymes required for its production were found here: *PGM2* (YMR105C, limS and limN) and *PRS4* (YBL068W, limN).

In pyrimidine metabolism, all enzymes for which orotate is a metabolite were discovered in our screens. Ura1p (YKL216W, limN) converts between orotate and dihydroorotate, while paralogs Ura5p (YML106W, limS) and Ura10p (YMR271C, limN) convert between orotate and orotidine-5P (Denis-Duphil 1989). The necessity of *URA5* and *URA10*, albeit under different starvation conditions, is one of the few instances where both paralogs were found to be of necessity for growth in at least one of our nutrient-limiting conditions. We acknowledge that the adjacency of these reactions to Ura3p [orotidine 5-monophosphate (OMP) decarboxylase], which converts orotidine-5P to uridine monophosphate, may be the reason for the necessity of these 3 genes when placed under stress. The strains screened were *ura3* auxotrophs, and while an abundance of uracil was provided, these 3 knockout strains demonstrated reduced growth in one of the 2 limiting conditions. Since OMP decarboxylase is irreversible under physiological *conditions* (Chan *et al*. 2009), it seems that the effect is limited to orotate need rather than the need for uridine phosphates.

### Role of carbohydrate metabolism

A considerable number of proteins acting in central metabolism pathways were found in our screens, with 20% of the genes tested of both glycolysis/gluconeogenesis (10) and citrate cycle (20) found in at least one screen. Many of these proteins act in more than one pathway as their metabolites are interchanged between pathways. Within many of these pathways, proteins encoded by genes we identified in these screens functioned in proximity rather than having functions scattered throughout the pathway. This bolsters the idea that particular subpathways or that the generation of or breakdown of particular metabolites is important for the cell during times of insufficient sulfur or nitrogen. Although we supplied cells with a standard concentration of glucose, perhaps reducing these other nutrients put enough stress on the cell that, in combination with a gene deletion that was otherwise survivable, cell concentration was negatively affected. Of course, amino acid metabolisms, previously discussed, are immediately adjacent to central metabolism, particularly to the citrate cycle.

In glycolysis/gluconeogenesis (10), most proteins we found to be required under limited nutrient conditions catalyze reactions involving α-glucose-6P. *GLK1* (YCL040W, limS and limN) encodes a hexokinase/glucokinase, catalyzing the reactions between α- and β-glucose to their 6P forms. Although *HXK2* encodes the main isoform of hexokinase in *S. cerevisiae* and its paralog *HXK1* encodes another isoform, neither of these 2 genes were found here. *YMR099C* (limS and limN) encodes hexose-6-phosphate mutarotase in *S. cerevisiae*, catalyzing the conversion between the α and β forms of glucose-6P as well as between that of galactose-6P and mannose-6P (Graille *et al*. 2006). Pgm2p (limS and limN) is a phosphoglucomutase, performing an important role in galactose utilization, among its activity in other metabolic pathways such as purine metabolism (230) in which it synthesizes PRPP. *pgm2Δ* cells have decreased glucose-1-phosphate, disrupting entry into glycolysis (Lin *et al*. 2015). Although it has 2 paralogs that were not found in our screens, Pgm2p appears to be important in both starvation conditions we tested. Another protein identified here, Tdh2p (YJR009C, limS), also has 2 paralogs that we did not find (Tdh1p and Tdh3p). These 3 proteins are each glyceraldehyde-3-phosphate dehydrogenases in *S. cerevisiae*. Although Tdh3p seems to have the same structure and function as Tdh2p (Delgado *et al*. 2001), it is not required for normal growth when sulfate is absent, suggesting a larger reliance on Tdh2p in this condition.

KEGG's glycolysis/gluconeogenesis pathway also includes the fate of pyruvate metabolic reactions. Thi3p (YDL080C, limN) converts pyruvate and thiamin pyrophosphate (TPP) to acetaldehyde, with the intermediary metabolite hydroxyethyl-TPP. While Thi3p acts as a TPP censor in the cell and, in low levels of thiamin, is required for its own gene expression as well as others (Nosaka *et al*. 2005), it was the only protein using thiamin or involved in its metabolism that we identified. *ADH6* (YMR318C, limS and limN) encodes an NADPH-dependent, medium chain, homodimeric alcohol dehydrogenase (ADH) with little specificity in terms of the alcohol and aldehyde substrates on which it acts (Larroy *et al*. 2002). Like most ADHs, Adh6p is involved in NAD+/NADP+ redox reactions. During these reactions, Adh6p reduces cinnamaldehyde, veratraldehyde, and furfural (Petersson *et al*. 2006). While important here in their conversion of acetate and acetaldehyde, both Ald3p (limS) and Ald4p (limS) were discussed above with the multiple amino acid metabolic pathways in which they also act.

Specific to pyruvate metabolism (620), *HFA1* (YMR207C, limS) encodes an acetyl-coenzyme A carboxylase required for fatty acid biosynthesis in mitochondria (Hoja *et al*. 2004). Cytochrome b2 (Cyb2p, YML054C, limN) is a lactate cytochrome oxidoreductase located in the intermembrane space of mitochondria. Expression of *CYB2* is repressed by glucose (Brown and Trumpower 1995). Several other genes identified in the pyruvate metabolism pathway have already been discussed: namely *LYS21* (220) and *MDH1* and *MDH3* (270). Along with *MDH1* and *MDH3*, *FUM1* and *ACS1* (below) are also of import not only to pyruvate metabolism but also to the citrate cycle.

Citrate cycle (20) proteins identified in our screens act adjacently and are located in the latter portion of the pathway, beginning with the reactant succinate and ending with citrate. *SDH4* (YDR178W, limN) and *SDH9* (YJL045W, limS) are genes encoding 2 of the 4 peptides comprising the succinate dehydrogenase tetramer, which converts succinate to fumarate, sending electrons to ubiquinol. Sdh4p, specifically, is 1 of 2 hydrophobic peptides within the enzyme, anchoring the complex to the membrane and binding to ubiquinone (Oyedotun and Lemire 1999). Along with Sdh3p (not found), Sdh4p contains the heme and quinone binding sites (Silkin *et al*. 2007). Sdh9p was previously thought to be associated with Sdh1p (not found), but a recent study has revealed that *SDH9* has a unique function and acts independently (Dickinson *et al*. 2022). *FUM1* (YPL262W, limS and limN) encodes fumarate hydratase, which converts fumarate to malate. Located throughout the cell in the mitochondrial matrix, cytosol, and nucleus, deletion or mutation of this gene has been found to cause a petit phenotype (Sass *et al*. 2003). In yeast, malate dehydrogenase has 3 isoforms, 2 of which were significant in these screens: *MDH1* (YKL085W, limS) encodes the mitochondrial malate dehydrogenase (Sumegi *et al*. 1992), while *MDH3* (YDL078C, limN) encodes that of the peroxisome (van Roermund *et al*. 1995). Mdh1p is part of the malate-aspartate NADH shuttle in mitochondria (Sumegi *et al*. 1992), and a deletion of *MDH3* results in reduced β-oxidation function (van Roermund *et al*. 1995). *CIT2* (YCR005C, limS and limN) encodes peroxisome citrate synthase. Its expression is decreased in the presence of inositol, tying its expression to phospholipid metabolism and signaling (564 and 4070) (Chen *et al*. 2002). Both yeast acetyl-CoA synthetase isoforms have the same enzymatic function, but when cells are grown on nonfermentable carbon sources, Acs1p (YAL054C, limS) produces most of the acetyl-CoA rather than Acs2p (Farrugia *et al*. 2019). Although not in a KEGG pathway, the mitochondrial malonate, oxaloacetate, and sulfate transporter, Oac1p (YKL120W, limS and limN), functions to bring these metabolites into the mitochondrial matrix (Palmieri *et al*. 1999).

In oxidative phosphorylation (190), components of complexes I, II, III, and IV and the ATPase were all found to be required for culture growth when cells were challenged with growth in the lack of sulfur or limited nitrogen. While we refer to “complex I” for simplicity, in reality, the NADH dehydrogenase acyl-carrier of *S. cerevisiae* is comprised of a single subunit: Acp1p, (not tested). Three genes encode associated NADH-ubiquinone reductases, of which *NDE1* (YDL085W, limS) and *NDI1* (YML120C, limN) were found in our screens. These, however, do not comprise complex I as seen in other eukaryotes but rather each have protein function as a single peptide (Timón-Gómez *et al*. 2018: 20). *SDH4* and *SDH9* encode subunits of complex II (succinate dehydrogenase) and therefore were discussed above with the citrate cycle (20). In complex III (the cytochrome-bc1 complex/cytochrome c reductase), Cor1p (YBL045C, limN) and Qcr2p/Cor2p (not found) dimerize to begin the assembly of the full complex (Stephan and Ott 2020). In addition, *QCR7* (YDR529C, limN) encodes an accessory protein of the complex and is required for usual rates of respiration (Malaney *et al*. 1997). A gene encoding cytochrome c, *CYC1* (YJR048W, limS) was found, although its paralog, *CYC7*, was not. Only one subunit of cytochrome c oxidase (complex IV) was found in our screens: *COX11* (YPL132W, limN). The additional stress of limited nutrients may have proved too much for strains with these electron transport chain (ETC) components deleted, while other components may be less important. (Timón-Gómez *et al*. 2018) have shown, however, that nitrogen starvation leads to the vacuolar degradation of mitochondria, with mitophagy specifically degrading complex I and complex III subunits, specifically Nde1p, Ndi1p, Cor1p, and Qcr2p (not tested). In addition to ETC subunits, 3 subunits of the ATPases were also identified: Vma5p (YKL080W, limS), a subunit of V-ATPase, and Atp7p (YKL016C, limN) and Atp18p (YML081C-A, limS and limN), both subunits of the F-type ATP-synthase.

In *S. cerevisiae*, KEGG lists 11 genes as encoding peptides involved in ascorbate and aldarate metabolism (53), all of which were tested. We identified 3 in each screen for a total of 5 of the 11. Three genes, *ALD3* (limS), *ALD4* (limS), and *ADH6* (limS and limN) have already been discussed, and their gene products function close to each other in this pathway. The other 2 were *ARA1* (YBR149W, limN) and *ALO1* (YML086C, limN). Ara1p, an NADPH-dependent arabinose dehydrogenase, and its NAD-dependent functional analog, Ara2p (not found) convert between arabinose and arabinono-1,4-lactone, although Hu *et al*. (2013) makes the case for the primary role of Ara1p to reduce dicarbonyl compounds that may accrue more readily during oxidative stress. Alo1p, an arabinono-1,4-lactone oxidase, then metabolizes the lactone to erythroascorbic acid, a compound important in oxidative stress response (Huh *et al*. 1998).

Outside of *PGM2* and *GLK1*, discussed above (10), 11 other genes involved in starch and sucrose metabolism (500) were determined to be required during sulfur and/or nitrogen starvation, comprising 36% of the peptides tested in the pathway. According to KEGG, there are 18 reactions catalyzed in *S. cerevisiae* within starch and sucrose metabolism, and those genes we found to be of import are involved in 13 of them (72%). Along with *PGM2* and *GLK1*, *FKS3* (YMR306W, limS and limN) was found in both screens. Fks3p is a 1,3-β-D-glucan synthase. It has been found that deletion of *FKS3* improves the normal stress resistance, viability, and antiautolytic abilities of the yeast cell (Wang *et al*. 2018). Of the 10 other genes observed to have an effect upon deletion, 6 were required under limited nitrogen conditions (*GDB1*, YPR184W; *GLG1*, YKR058W; *IMA1*, YGR287C; *IMA5*, YJL216C; *MAL12*, YGR292W; and *SPR1*, YOR190W), and 4 were required when sulfur was absent (*GPH1*, YPR160W; *SGA1*, YIL099W; *TPS3*, YMR261C; and *YHL012W*). Most of these genes have been shown to be of importance during starvation conditions, but thus far, primarily only for carbon shortages (François and Parrou 2001).

### Role of fatty acid and glycerolipid metabolism

Although several lipid metabolic pathways included some proteins that we determined to be essential for growth in nutrient starvation conditions, only one of these pathways stood out: fatty acid elongation (62) is another pathway with few members—only 8 in baker's yeast, with only 6 that were able to be tested here. Of these, we found that 4 were required for culture growth in either sulfur- or nitrogen-limiting media. Elo1p (YJL196C, limS and limN) and Elo3p (YLR372W, limS and limN), both elongases, were discovered to be of import in both screens. Elo1p and its paralog, Elo2p (not found), synthesize medium chain fatty acids, while Elo3p synthesizes very long chains (Klug and Daum 2014), with the next step in long chain acyl-CoA synthesis carried out by Ifa38p (YBR159W, limN) as a β-keto-reductase (Wang *et al*. 2018). Upon synthesis completion, Tes1p (YJR019C, limS), a thioesterase, can then convert them to long chain fatty acids. *TES1* expression increases upon growth on oleate (Jones *et al*. 1999) but has not yet been implicated in nutrient-limited cell needs.

Other metabolic pathways involving lipid components are grouped into “multimacromolecule” metabolisms (Fig. 1). Those found to be of note were glycerolipid (561), glycerophospholipid (564), and inositol phosphate (562) metabolic pathways.

Glycerolipid metabolism (561) is yet another pathway in which Ald3p, Ald4p, and Adh6p are involved. These 3 proteins participate in converting glycerate to glyceraldehyde to glycerol. There are 7 other genes found in our screens that also encode proteins that function here, making up 32% of the genes tested and affecting 9 of the 16 reactions that occur in *S. cerevisiae*. The interconversion between glycerol and glycerone is carried out by Gcy1p (YOR120W, limN) (Rippert *et al*. 2021) and from glycerone to glycerone phosphate by Dak1p (YML070W, limS) a dihydroxyacetone kinase. Although Dak1p and Dak2p (not found) phosphorylate the toxic dihydroxyacetone (Molin *et al*. 2003), here Dak1p may be phosphorylating glycerone, creating a reactant for glycolysis or glycerophospholipid (564) anabolism, whereas in other pathways, it may act to generate glyceraldehyde-3P (G3P), again, to provide entry of carbon into glycolysis. Gut1p (YHL032C, limN) is another kinase, and this one phosphorylates glycerol during phospholipid synthesis. It is 1 of 3 proteins primarily associated with the glycerol catabolism pathway, the other 2 being Gut2p, the mitochondrial kinase, and Stl1p, the glycerol plasma membrane permease, (Swinnen *et al*. 2013), and neither of which were found here, suggesting that it is not likely be glycerol itself that is important in nutrient starvation but rather a more general stress on metabolism that is in effect. Lro1p (YNR008W, limS and limN) is a diacylglycerol (DAG) acyltransferase found to reside in both the endoplasmic reticulum (ER) and nuclear membranes of yeast, converting phospholipid-derived fatty acids into triacylglycerols. Barbosa *et al*. (2019) showed that Lro1p is most active when cells are faced with starvation conditions, namely not only when grown without a carbon source but also when grown in a 16% reduction in YNB with ammonium sulfate. Here, we found it was vital for normal culture growth when grown in the absence of either a nitrogen or sulfur source. Tgl2p (YDR058C, limS and limN) is a purported yeast di-/triacylglycerol lipase likely acting on DAG (Odendall *et al*. 2019). Odendall *et al*. (2019) also identified *CQD2*/*MCP2* (YLR253W, limS) to negatively interact with *TGL2*, suggesting that without these 2 genes, DAG builds up in mitochondria, leading to toxicity. Lro1p and Tgl2p catalyze opposing reactions, and both were found in both screens, suggesting the importance of DAG/triacylglycerol interconversion in starvation. Finally, the lysophospholipid acyltransferases Ale1p (YOR175C, limN) and Loa1p (YPR139C, limN) were both found to be required when yeast were grown in limiting nitrogen conditions. These enzymes join lysophosphatidic acid to acyl-CoA, yielding phosphatidic acid (Ayciriex *et al*. 2012; Morisada *et al*. 2018).

Ale1p and Loa1p both also act in glycerophospholipid metabolism (564). Forty percent of genes involved in this pathway that were tested were found to be vital in at least one of our screens, with 23% required when starved for sulfur and 29% found when starved for nitrogen. A key metabolite of the pathway is phosphatidylglycerol (PG), and several genes found here encode proteins working with or adjacent to the compound. Both Gep4p (YHR100C, limS) and Ale1p (561 above) synthesize PG, while Pgc1p (YPL206C, limN) and Crd1p (YDL142C, limS and limN) use PG as a substrate. Gep4p is a mitochondrial phosphatidylglycerophosphatase involved in the cardiolipin synthetic pathway and is found on the inner membrane (Osman *et al*. 2010). PG phospholipase C (Pgc1p) degrades PG by cleaving it into DAG and glycerophosphate (Pokorná *et al*. 2016). Pgc1p is located primarily in lipid droplets and the mitochondria. Crd1p is a cardiolipin synthase and, like Gep4p, is found on the inner membrane. When its expression is repressed, the amount of acetyl-CoA is also lessened (Pokorná *et al*. 2016). Cardiolipin is found in the inner membrane of mitochondria and is necessary for the citric acid cycle to function normally (Raja *et al*. 2019). The mitochondrial proteins Cld1p (YGR110W, limS) and Taz1p (YPR140W, limN) carry out opposing reactions in the conversion between cardiolipin and monolysocardiolipin. Cld1p is a cardiolipin-specific phospholipase, while Taz1p is a lyso-phosphatidylcholine acyltransferase that synthesizes cardiolipin (Testet *et al*. 2005). Genes encoding several other phospholipases involved in the degradation of phosphatidyl compounds were found here. *SPO14* (YKR031C, limS and limN) encodes phospholipase D, which hydrolyzes phosphatidylcholine into choline and phosphatidic acid (Sreenivas *et al*. 1998). We also found several phospholipase Bs: Nte1p (YML059C, limS and limN), which is phosphatidylcholine-specific; Plb1p (YMR008C, limS); and Plb2p (YMR006C, limS and limN) (Merkel *et al*. 2005). In addition, we identified several kinases involved in phosphatidyl synthesis, namely Eki1p (YDR147W, limN), an ethanolamine kinase that catalyzes the committed step in phosphatidylethanolamine synthesis (Kersting and Carman 2006); Cki1p (YLR133W, limN), a choline kinase involved in both phosphatidylcholine and phosphatidylethanolamine synthesis (Dowd *et al*. 2001); and Dgk1p (YOR311C, limS), a DAG kinase.

Proteins functioning in inositol phosphate metabolism (562) were also discovered here, but not those that immediately lead to the glycerophospholipid metabolism pathway. Rather, phosphatases directly regenerating inositol were found—Inm1p (YHR046C, limS and limN) dephosphorylates IP, from the 1, 3, and 4 carbon (Klug and Daum 2014), and both Inp52p (YNL106C, limS) and Inp53p (YOR109W, limN) dephosphorylate PI(4,5)P_2_ at carbon 5. We see these phosphatases again in the phosphatidylinositol (PI) signaling system (4070) below.

### Role of vitamin metabolic pathways

YNB is the SD medium that has been widely adopted by the yeast community, with a standard amount of ammonium sulfate and glucose normally added. When Wickerham (Wickerham and Burton 1948; Wickerham 1951) devised his classic defined media in order to taxonomically classify yeasts, he included the B vitamins of the time, and these remain in the formulation of YNB today, even though *S. cerevisiae* can make these compounds. Although niacin (B3), pyridoxine (B6), and biotin (B7) were all supplied in the growth media, a substantial number of genes encoding enzymes in the metabolic pathways of these vitamins and their metabolites (vitamers) were found here to be important for growth when sulfur and/or nitrogen was limited. Of the pathways for which we identified no genes in our screens, many were that of other B vitamins, which were also supplied [B1, thiamin (730); B2, riboflavin (740); B9, folic acid; and B10, p-aminobenzoic acid (790)]. There were only 2 other B vitamins included in the media—one was B5 (pantothenate), which is generated both in β-alanine metabolism (410 above) and in its own pathway (770), in which only Ilv6p (YCL009C, limN), an enzyme that acts on pyruvate, and both of the sulfur-identified proteins Ald3p and Ald4p were found. The other was the historically named B8, which is inositol.

Several proteins in nicotinate and nicotinamide metabolism (760) were detected in the nitrogen screen and one in the sulfur screen. *NMA1* (YLR328W, limN) encodes nicotinamide mononucleotide (NMN) adenylyltransferase, which is a part of the NAD biosynthesis pathway. Nma1p produces NAD by catalyzing the addition of an adenylyl group to NMN (Emanuelli *et al*. 1999). Pof1p (YCL047C, limN) appears to act as an NMN adenylyltransferase that, unlike Nma1p, specifically targets NMN. In *pof1Δ* strains, NAD levels are significantly decreased. Other effects of *pof1Δ* include a decrease in nicotinamide riboside utilization efficiency and a decrease in oxidative stress resistance (Kato and Lin 2014). Nrk1p (YNL129W, limS) is a kinase that phosphorylates nicotinamide riboside thereby producing NMN (McClure *et al*. 2008). Here, *NRK1* was not identified as being important when starved for nitrogen, but it was important in sulfur starvation conditions.

Also falling into nicotinate and nicotinamide metabolism were 2 histone deacetylases. The NAD-dependent deacetylase Sir2p (YDL042C, limN) targets histone tails (Humphrey *et al*. 2020). Sir2p functions to silence telomeres, thereby protecting the integrity of the genome and reducing the effects of aging. In addition to targeting histones, Sir2p is believed to deacetylate proteins with roles in metabolism and the aging processes (Vall-Llaura *et al*. 2019), and it has been found to be activated by an increased NAD in the cytosol provided by the malate-aspartate NADH shuttle comprised of Aat1p and Mdh1p (Easlon *et al*. 2008), both found to be of importance in our screens and discussed above with amino acid metabolism and the citric acid cycle. *HST3* (YOR025W, limN) encodes NAD-dependent histone deacetylase, and thereby it is also involved in nucleic acid metabolism and the assembly of subtelomeric heterochromatin. Starting in late S phase and functioning through G2/M phase, Hst3p catalyzes the deacetylation of H3 at position K56. It has been found that *hst3Δ* strains have shorter lifespans, which is theorized to be the result of genomic instability (Hachinohe *et al*. 2011).

A substantial number of enzymes involved in vitamin B6 (pyridoxine) metabolism (750) were found in both screens. *PDX3* (YBR035C, limS) encodes pyridoxine/pyridoxamine 5′-phosphate oxidase, though little has been described about its full role since its discovery (Loubbardi *et al*. 1995). The product encoded by *YPR127W* (limS and limN), while uncharacterized, is homologous to a pyridoxine/pyridoxamine 5′-phosphate oxidase in *Schizosaccharomyces pombe* (Zuzuarregui *et al*. 2006). *SNZ1* (YMR096W, limS and limN), *SNZ2* (YNL333W, limN), and *SNZ3* (not tested) are paralogs that encode pyridoxal phosphate (PLP) synthases and lead from pyridoxine metabolism to that of thiamin (Paxhia and Downs 2019). *SNO1* (YMR095C, limS and limN) interacts with both *SNZ1* and *SNZ2* in the synthesis of PLP from glutamine (Padilla *et al*. 1998).

The synthesis of dethiobiotin, the immediate precursor of biotin (780), seemed to be important to growth when nutrients were limited. Although the enzyme that metabolizes it into biotin, Bio2p, was not found to be required, both enzymes that synthesize dethiobiotin from 8-amino-7-oxononanoate were. The deletion of both *BIO3* (YNR058W, limS and limN) and *BIO4* (YNR057C, limS) negatively affected cell culture growth in limiting conditions. Although not listed in this KEGG pathway, Bio5p (YNR056C, limS), the plasma membrane-localized 8-amino-7-oxononanoate permease (Phalip *et al*. 1999) was also required for growth when sulfur was lacking. Although these genes have been found to be of importance in nitrogen-deficient conditions (of ∼80% that of control) (Brice *et al*. 2014), less is known about their role in sulfur starvation.

When these vitamins (B3, B6, and B7) were supplied, why were so many proteins involved in their metabolisms required for growth in limited nutrient media? We suggest that their use is critical to the cell as it mounts a response to these starvation conditions. With NAD performing a role in central metabolism, its precursor niacin (nicotinic acid, B3) is critical for cell survival. NAD is also a cofactor for an abundance of oxidoreductases and deacetylases as well as a precursor of NADP. Perli *et al*. (2020) compiled those *S. cerevisiae* proteins for which vitamins B1, B6, and B7 are either a coenzyme or a substrate. Pyridoxine (B6) and its vitamers affect the most enzymes by far, with 50 proteins known to use them, and functioning in the metabolisms of carbohydrates, amino acids, and lipids. Of these 50, genes encoding 16 of them were found to be important during sulfur and/or nitrogen starvation here (*AAT1*, *ALT1*, *ARO8*, *BIO3*, *CHA1*, *CYS3*, *DSD1*, *GAD1*, *GCV2*, *GPH1*, *IRC7*, *SHM2*, *SRY1*, *TRP5*, *YHR112C*, and *YML082W*), and we found 45% of the proteins which function in pyridoxine metabolism to be of importance during starvation. Perli *et al.* (2020) only lists 6 proteins as using biotin (B7), and we observed the importance of 2 in our screens (*DUR12*, in arginine biosynthesis, and *HFA1*, YMR207C, limS, in fatty acid metabolism). We identified 40% of those proteins functioning in biotin metabolism to be crucial during starvation. Of the 12 proteins Perli *et al.* (2020) note that require thiamin (B1), only one was identified here (*THI3*, in glycolysis), and we found that no gene we tested in thiamin biosynthesis to be critical to cell growth during starvation. Stated together, for those metabolic pathways of vitamins that we found most crucial under nutrient-limiting conditions, we observed a need for a larger proportion of proteins that use those vitamins and vitamers as coenzymes, underlining the need for a supply of the coenzyme for the maintaining of enzyme functionality.

### Cellular processes and signaling pathways

We have grouped the KEGG signaling pathways and cellular processes discussed below into ribosomes; mitogen-activated protein kinase (MAPK) signaling; nuclear division, covering mitosis and meiosis proteins; PI signaling and vesicle fusion; pathways targeting the ER; autophagy and mitophagy; and various endocytic pathways (Fig. 1; Supplementary Table 3). More than in our discussion of metabolic pathways, here we note a number of identified proteins with functions related to these signaling paths that are not currently noted in the KEGG pathway database.

### Role of the ribosome

The yeast ribosome (3010) is composed of 2 large subunits, 60S and 40S, consisting of 79 peptides. Many of the peptides are duplicates that share identical or nearly identical function, often denoted with an A and B naming system. When the individual genes encoding these peptides are deleted, duplicated, or not, there are a variety of different severities. The duplication of the genes allows for greater chances of survival under stress and allows for the subfunctionality and regulation of each gene (Parenteau *et al*. 2015). Indeed in most cases, the genes we observed as required are 1 of 2 genes resulting from a duplication event, with the deletion of one paralog causing the cell to succumb to the stress of limited nutrients, while the deletion of the other counterpart could overcome this stress. This may be similar to what Evangelisti and Conant (2010) marked of ribosomal peptides, with 1 of 2 paralogs being essential for a particular function while the other paralog was not. Ni and Snyder (2001) screened a diploid yeast deletion library and identified ribosomal components required for normal bud localization. Of the 15 ribosomal gene deletants they found with a strong phenotype, we tested 11 and found 3 (*RPS7A*, YOR096W, limS and limN; *RPS29A*, YLR388W, limS; and *RPS1B*, YML063W, limN). Like Ni and Snyder, in each of these cases, we did not find the paralog to have an effect. We did, however, find a paralog of one of their ribosomal genes to be required for normal growth but not the isoform they discovered to be of importance in bud formation: *RPL14B* (YHL001W, limN).

### Role of MAPK signaling

KEGG delineates 4 MAPK signaling pathways in yeast (4011) by type: pheromone, high osmolarity, cell wall stress, and starvation. Proteins in the middle of both the pheromone and cell wall stress pathways effect actin polarization, but the genes encoding those particular proteins were not discovered in our screens. KEGG reports that all of the yeast MAPK pathways except starvation feed directly into their cell cycle pathway (4111); starvation results in filamentation. Although starvation itself will directly impact the cell cycle (see our findings below), the yeast MAPK starvation signaling pathway does not directly signal to proteins involved in the cell cycle KEGG pathway. Several signaling proteins, including some found in our screens, are present in more than one yeast MAPK pathway except for cell wall stress, which has no proteins in common with the other 3. Here, we group genes found in our screens by these 4 signaling pathways.

Early in the pheromone signaling pathways, Yck2p (YNL154C, limS and limN), a casein kinase (CK1), phosphorylates Ste2p and Ste3p to downregulate their plasma membrane expression (Hicke *et al*. 1998). Interestingly, this kinase and its paralog have also been found to function in sensing glucose (Snowdon and Johnston 2016). Its significance in growth in nitrogen- and in sulfur-limited environments suggests additional roles or implies that when under general starvation, there is an increased need for glucose detection. *BEM1* (YBR200W, limN) encodes a scaffolding protein with an SH3 domain used in this pheromone response cascade, but it also contains a PI(3)P-binding domain (Marat and Haucke 2016). Bem1p has been shown to not only bind to kinases found in pheromone signaling but also to monomeric GTPases and their associated proteins involved in bud formation and has been shown to be crucial itself in bud localization (Madden and Snyder 1998). Binding to Bem1p, Cla4p (YNL298W, limN sulfur) is a p21-activated kinase family member involved upstream of several cellular processes, including cell polarization, vacuole inheritance, and macromolecule metabolic processes such as sterol uptake. *cla4Δ* cells are unable to regulate their mitotic exit network and morphogenesis through this pathway (Wild *et al*. 2004), but Cla4p seems to be independent of a MAPK pathway when regulating sterol uptake (Beranek *et al*. 2009). One of its targets is Ste11p, which is itself a MAPKKK. These 2 kinases are also active in high osmolarity signaling (Westfall *et al*. 2004), and Ste11p is active in starvation signaling. *DIG1* (YPL049C, limN) and *DIG2* (YDR480W, limN) were both found to be required in limited nitrogen. These 2 paralogs are inhibited by this MAPK cascade, themselves acting as inhibitors of Ste12p (not found), regulating the invasive growth response in glucose-limited conditions through independent mechanisms (Olson *et al*. 2000) and are also active in starvation signaling. Without their inhibition, the cell would move into cell cycle arrest. At the end of the pheromone signaling pathway, Cln1p (YMR199W, limS and limN) is normally inhibited to initiate cell cycle arrest. It is a G1/S-specific cyclin that aids in the activation of the mitotic cell cycle. Despite its importance, *CLN1* is not only essential for standard laboratory growth in *S. cerevisiae* but also has been found to be nonessential under normal conditions upon deletion in another yeast species, *Cryptococcus neoformans* (Baroni *et al*. 1994; Altamirano *et al*. 2021). Here, however, it seems that when the cell is starved for nitrogen, the deletion of *CLN1* is too much stress for cell survival. Although the haploid cells studied in these screens are not exposed to mating pheromones and therefore should not be transducing signal from them, Williams *et al*. (2016) have explored the idea that pheromone response and response to carbon and nitrogen starvation have overlapping phenotypes including the expression profile of some genes. Perhaps in addition, there is some overlap in the signaling system between pheromone and starvation response that facilitates this phenotype.

The high osmolarity signaling pathway employs 2 proteins also acting in the middle of the pheromone pathways: Cla4p and Ste11p. Here, the MAPKKK Ste11p and Ssk22p (YCR073C, limN) phosphorylate the MAPKK Pbs2p (not found) on its N-terminal region (Tatebayashi *et al*. 2003). Pbs2p then phosphorylates Hog1p (YLR113W, limN), a stress-activated MAPK that then phosphorylates a variety of proteins involved in various aspects of osmolyte synthesis (Tognetti *et al*. 2020), including Sko1p (YNL167C, limS and limN), Sic1p (YLR079W, limS and limN), and Hsl1p (YKL101W, limS). Sko1p is an activating transcription factors/cAMP response element binding protein (ATF/CREB) transcription factor involved in both positive and negative transcriptional regulation under stress (Sri Theivakadadcham *et al*. 2019). Sic1p is a cyclin-dependent kinase (CDK) inhibitor, which is positively affected by its phosphorylation by Hog1p (Clotet *et al*. 2006) and, when active, inhibits Clb5p (YPR120C, limN), a B-type cyclin relied upon during S phase (McCune *et al*. 2008). Hog1p also phosphorylates the septin-binding kinase Hsl1p (YKL101W, limS), inhibiting it and thereby negatively affecting bud neck formation (Clotet *et al*. 2006). The signals transmitted by Hog1p are abbreviated by Ptc1p (YDL006W, limS and limN), a type 2C protein phosphatase that dephosphorylates Hog1p. The deletion of *PTC1* delays the movement of mitochondria across the bud neck (Roeder *et al*. 1998), which may have an important, negative effect on growth when cells are lacking sulfur or nitrogen, particularly given how important the activity of mitochondrial components is as shown in our screens. Ubp5p (YER144C, limN), a putative ubiquitin-specific protease that KEGG places in endocytosis (4144), also seems to have effects in the formation of the bud neck (Amerik *et al*. 2006).

One of the proteins that senses cell wall stress is the plasma membrane protein Mid2p (YLR332W, limS and limN) (Green *et al*. 2003). The stress signal is transduced through a signaling pathway, at the end of which is found a MAPK cascade (Ribeiro *et al*. 2022). Mkk2p (YPL140C, limS) is a MAPKK, which phosphorylates Slt2p (YHR030C, limS), and a MAPK, which phosphorylates Rlm1p (YPL089C, limN). Sdp1p (YIL113W, limS) is a tyrosine protein phosphatase, which dephosphorylates Slt2p, turning off the signal. Rlm1p is a MADS-box transcription factor that binds to DNA to effect the structural integrity and maintenance of the cell wall, proving a high value to the vitality of cells under normal circumstances (Terzioğlu *et al*. 2020), but more so when cells are placed under stress. The interconnectedness of these proteins required for growth under limited nutrient conditions accentuates the importance of cell wall maintenance and the stress put upon it during times of nutrient starvation. Swi6p (YLR182W, limS and limN) is a transcriptional regulator involved in the G1/S transition of the cell cycle (discussed below), particularly during heat-induced stress. Swi2p also affects the unfolded protein response in the ER lumen, the signal transduced to the cytoplasm upon unfolded proteins binding to Ire1p (YHR079C, limS) (Scrimale *et al*. 2009). The pathway culminates in the upregulation of *FKS3* (YMR306W, limS and limN), which is involved in cell wall remodeling and aiding the cell in stress resistance and antiautolytic ability by delaying the cell's response to its environment (Wang *et al*. 2018).

The last MAPK signal path delineated in KEGG, the starvation signaling pathway, also uses the MAPK Ste11p and the MAPK-responsive inhibitors Dig1p and Dig2p discussed above. Unique to this MAPK pathway is *MSS11* (YMR164C, limS and limN), encoding a transcription factor. Activity of Mss11p implies, however, that the protein resides at the crossing of 2 different signaling cascades—the one for filamentous growth and the other affecting starch metabolism (Gagiano *et al*. 2003). Additionally, Mss11p has been shown to be required for the expression of the essential gene *STA2* (not tested) in reduced ammonium media (Gagiano *et al*. 2003). The glucoamylase Sta2p is required for normal laboratory growth. With the importance of carbohydrate metabolism under sulfur and nitrogen starvation conditions that we have observed, it can be no surprise that deleting a transcription factor responsible for its expression under low nitrogen conditions would negatively affect culture growth.

### Role of nuclear division

Although the mitotic cell cycle (4111) and meiosis (4113) in yeast are controlled separately, there are several genes that we found to be of importance that function in both processes. Here, we discuss a fraction of the proteins found here to be required during starvation in these processes. KEGG notes Sic1p and Clb5p in both nuclear division processes and both were discussed in high osmolarity MAPK signaling above. Sic1p is a CDK inhibitor that acts on Cdk1/Clb complexes, including that of the cyclin Clb5p, which drive the progression of the cell cycle from G1 into S phase. Sic1p also plays a role in regulating the cell's exit of mitosis (Barberis *et al*. 2012). The cyclin Clb5p is present in abundance from the start of the S phase until the end of anaphase, when the anaphase promoting complex (APC/C), an ubiquitin-protein ligase, tags them for degradation (McCune *et al*. 2008). Two subunits of that complex, Cdc26p (YFR036W, limS and limN) and Apc9p (YLR102C, limN), were found in our screens. Cdc26p may be responsible for the stabilization of the APC/C, particularly when the cell is under stress (Hwang and Murray 1997), and when yeast cells are under stress, *APC9* expression is lowered, which may prevent cell division (Peña *et al*. 2015), pointing to the importance of a fully functional APC/C during nutrient starvation.

Ime2p (YJL106W, limN) is a serine/threonine protein kinase that sits at the center of meiosis regulation (Schindler and Winter 2006) with the master regulator of meiosis Ime1p (not found). Ime2p is purportedly only relevant to meiosis, in that it regulates the stability of Ime1p, but given that Ime1p was not also found suggests that Ime2p may have an additional role or that the transcription factor Ime1p is not the master regulator as we understand it (it is not, after all, essential for growth in standard laboratory conditions). In early meiosis, Ime2p phosphorylates Sic1p, which relieves the inhibition of Clb5p, promoting the G_1_/S transition. Later in meiosis, Ime2p is necessary for the G_2_/M transition and chromosome separation (Benjamin *et al*. 2003). *IME2* and 9 other genes we found to be essential to cell growth when starved for sulfur or nitrogen are only indicated in the meiotic process. Their discovery here suggests a potential role they may have in other processes, since these screens were conducted in haploid cells.

Swi6p (YLR182W, limS and limN), mentioned in cell wall MAPK signaling above, is a transcriptional regulator that is part of 2 binding factors: SBF, Swi6p complexed with Swi4p (not found), and MBF, Swi6p complexed with Mbp1p (YDL056W, limN). Both binding factors promote progression into S phase (Taylor *et al*. 2000), but MBF specifically binds to the MluI cell cycle box regulatory element, which is critical in genes expressed for regulation of the G1/S checkpoint (Bean *et al*. 2005). Spo12p (YHR152W, limS) and Slk19p (YOR195W, limN) complex with other peptides to establish the CDC fourteen early anaphase release (FEAR) complex, the activity of which is required for exit from mitosis and meiosis I (Stern 2003). Both Spo12p and Slk19p inhibit inhibitors of the essential protein Net1p (not tested), which works with the essential protein Cdc14p (not tested), required for telophase exit. Bfa1p (YJR053W, limS), involved in mitotic spindle alignment (Lee *et al*. 1999), signals to Dbf20p (YPR111W, limN), which phosphorylates Cdc14p. The requirement of these mitotic exit network members when cells are grown in limited nutrient conditions may be due to effects further upstream of nuclear division. Many of the proteins used during nuclear division are outright essential (around 40%). While those we found in our screens are not required for growth in a rich medium, the pressures of resource scarcity may have been enough to convert them to essential genes, causing these 2 pathways to be affected.

### Role of PI signaling and vesicle fusion

Intracellular trafficking is crucial for cells to transport proteins to the correct membrane or lumen for proper maturation and function; for cells to bring in extracellular molecules for nutrition or environmental response; or to downregulate and/or recycle receptors, membrane components, or excessive or damaged intracellular components. Most cellular membranes are defined by the PIs that comprise them. While not found in abundance, PI and its various phosphorylated states mark membranes, distinguishing the particular membrane of one compartment from others within the cell. As these lipids move from one compartment to another as compartments pinch off and merge, specific kinases and phosphatases maintain the phosphorylation state of localized PIs/phosphorylated counterparts (PIPs). Thus, the PI signaling system (4070) is important in delineating compartments. Here, we found over 30% of the members of this system in our screens.

Vip1p (YLR410W, limN) and Kcs1p (YDR017C, limN) are both inositol polyphosphate kinases, the first being the kinase that phosphorylates IP_6_ (phytic acid) to 1PP-IP_5_ and 5PP-IP_5_ to IP_8_ and the latter being that which phosphorylates IP_6_ to 5PP-IP_5_ and 1PP-IP_5_ to IP_8_. These kinases have functions distinct from each other (Bhandari *et al*. 2007), and the fact that both are crucial during nitrogen starvation strongly implicates the importance of IP_8_ in cellular response, whether it be the presence of IP_8_ or in turning down or off the signal presented by IP_6_ and the PP-IP_5_s. It has been noted that the absence of these kinases results in extensive vesicular trafficking dysregulation (Bennett *et al*. 2006). We also found Pho84p (YML123C, limN), an inorganic phosphate transporter that is upregulated when cells are isopentenyl pyrophosphate (IPP) deficient and in *kcs1Δ* cells (Bennett *et al*. 2006). Although not noted in a KEGG pathway, Pho84p is involved in the PI signaling process.

Both polyphosphatidylinositol phosphatase paralogs, Inp52p (YNL106C, limS) and Inp53p (YOR109W, limN), were found in one screen, but each was important in different conditions. Both dephosphorylate PI(4,5)P_2_ to PI(4)P and PI(3,4,5)P_3_ to PI(3,4)P_2_. While the other isoforms of PI phosphatase were not found in these screens (Inp51p and Inp54p), they are both PI 4,5-bisphosphate 5-phosphatases and only dephosphorylate a single substrate (Strahl and Thorner 2007). Those we identified to be required for normal growth in the absence of a nitrogen source, Inp52p and Inp53p, have a wider range of substrates and therefore are able to dephosphorylate most phosphoinositides, thereby turning off any PI phosphorylation signal or assisting in the maintenance of a PIP variant specific to a particular cellular compartment (Klug and Daum 2014). Indeed, their double deletion negatively affects receptor-mediated endocytosis and endosomal sorting of clathrin-coated vesicles, and Inp53p is implicated in cytosol to vacuole targeting, a type of autophagy (Strahl and Thorner 2007). Both Inp52p and Inp53p have also been shown to interact with Bsp1p (YRP171W, limS), an actin-binding protein not appearing in a KEGG pathway, whereas Inp51p does not (Strahl and Thorner 2007). Another phosphatase, Inm1p (YHR046C, limS and limN), an inositol monophosphatase that dephosphorylates I(1)P, I(3)P, and I(4)P to inositol (I), is also identified here.

When yeast cells are faced with a lack of sulfur, vesicular movement plays an important role that cannot be understated. In the limited sulfur screen, 40% of the total peptides from tested genes were involved in SNARE interactions in vesicular transport (4130). SNAREs are peptides that complex in order to facilitate fusion between vesicles and their target membranes and are frequently categorized as being found on the vesicle (v-SNARE) or on the target (t-SNARE). Vam7p (YGL212W, limS), a vacuolar t-SNARE (Lee *et al*. 2006), is also found in autophagy (4138). Sso2p (YMR183C, limS and limN) and Tlg2p (YOL018C, limS), both syntaxin homologs, are also t-SNAREs, with Sso2p localized to the plasma membrane (Peer *et al*. 2022) and Tlg2p functioning in endocytosis and in the trans-Golgi network (Toshima *et al*. 2023). The only v-SNARE identified in the screens was Snc2p (YOR327C, limS and limN), which works in the plasma membrane (Shen *et al*. 2013). Those SNAREs we found function from the trans-Golgi network outward; we did not find any SNAREs functioning between the ER through to the Golgi; however, only 2 of the 10 are testable.

### Role of ER targeting pathways

KEGG simply calls the process by which proteins are targeted to the ER “protein export” (3060), perhaps because orthologs and functional analogs are present in prokaryotes. Here, an astounding 52% of the genes tested in this process were determined to be crucial for growth under starvation conditions. In the Sec-dependent pathway, Sec61 is the translocator complex, facilitating the insertion of a majority of peptides into the ER membrane or lumen and, in yeast, has 3 subunits. Sec61p, a SEC61α and the pore proper, is essential and therefore was not tested; its paralog, the SEC61α Ssh1p (YBR283C, limS), however, was identified here and has been found to act independently from Sec61p (Wilkinson *et al*. 2001). Two SEC61βs are present in the genome, one of which, Sbh1p (YER087C-B, limS), was statistically relevant here, and although little is currently known about its function, it is regulated by Ess1p (not tested) (Barbieri *et al*. 2023). The genes encoding the only SEC61γ in *S. cerevisiae* (*SSS1*), the 6 subunits of the signal recognition particle (SRP), and the 2 SRP receptor subunits were all essential and therefore not tested.

In the guided entry of tail-anchored protein (GET) pathway, Get1p (YGL020C, limS) is one of a set of heterodimers comprising the insertase responsible for inserting SNAREs into the membrane (Wang *et al*. 2014). We have already noted above the importance of SNAREs during conditions of nutrient shortage, and Get1p is also involved in amino acid metabolisms discussed above (260 and 400). The GET pathway targeting factor, Get3p (YDL100C, limS and limN) was also required when cells were starved and has been shown to be crucial under oxidative stress (Voth *et al*. 2014). The GET pathway as a whole is important in proteostasis management (Josefson *et al*. 2023).

A third, “backup” ER targeting process, the SRP-independent pathway, involves 3 peptides, 2 of which were found here: Snd1p (YDR186C, limN), the targeting factor, and Snd2p (YLR065C, limS and limN), one of the heterodimerizing translocators (Aviram *et al*. 2016). Peptides translocated to the ER often are targeted through a signal peptide that may be cleaved. The yeast signal peptidase tetramer was required for growth in our screens: signal peptidase complex subunits Spc1p (YJR010C-A, limS) and Spc2p (YML055W, limN). The other 2 subunits, Spc3p and Sec11p, are essential and therefore were not tested. Little work has been done on the precise function of either Spc1p or Spc2p, but it appears that they regulate the process and level of glycosylation (Hosomi *et al*. 2020). In the inner membrane of mitochondria, another signal peptidase, IMP, carries out signal peptide cleavage (Luo *et al*. 2006). One of the 2 catalytic subunits, Imp1p (YMR150C, limN), was found in our screen.

### Role of autophagic pathways

In *S. cerevisiae*, autophagy (4136) and mitophagy (4139) (Supplementary Figs. 1 and 2) are distinct processes, but Atg11p (YPR049C, limS) is a scaffolding protein at the center of both (Yang and Klionsky 2009). Through its interactions with the receptors Atg13p (YPR185W, limS and limN; autophagy) and Atg32p (YIL146C, limS; mitophagy), Atg11p activates selective phagophore assembly sites (Zientara-Rytter and Subramani 2020). Atg13p is the regulatory subunit of the initiating ATG1 complex (comprised of Atg11p and Atg13p) that receives the autophagy signal during starvation (Reggiori *et al*. 2004). Atg32p sits at the center of mitophagy, serving as the predominant regulator of the event (Okamoto *et al*. 2009). Atg32p integrates signals from several other pathways. Upstream of both the ATG1 complex in autophagy and Atg32p in mitophagy is Tor1p (YJR066W, limN), a kinase whose inactivation during nutrient starvation conditions can lead to G_1_ arrest, thereby also positioned as an upstream signal for meiosis (4113). It has been shown that Tor1p assists in inducing autophagy in yeast undergoing starvation (Noda and Ohsumi 1998), and its presence destabilizes the interaction between Atg32p and Atg11p, preventing mitophagy (Innokentev and Kanki 2021). In mammals, it has been found that phospholipase D is required for mTORC activation in autophagy (Marat and Haucke 2016). Here, we found *S. cerevisiae*'s phospholipase D, Spo14p (limS and limN), to be of consequence. Unfortunately for our screens here, several members of the target of rapamycin (TOR) complexes in yeast (González and Hall 2017) are essential and were therefore not included in the knockout library: Tor2p (paralog of Tor1p), Kog1p (subunit of TORC1, rapamycin-sensitive complex), Snf1p [adenosine monophosphate (AMP)-activated protein kinase (AMPK), phosphorylates Kcs1p, discussed in PI signaling (4070)], and Lst8p (subunit of TORC1/2).

In addition to Tor1p, phosphokinase A (PKA) has been shown to regulate autophagy during stress in yeast, particularly during starvation (Boer *et al*. 2008). PKA has an inhibitory effect on Atg13p, and the MAPK Slt2p (limS) inhibits PKA by phosphorylation. Surprisingly, we did not find any of the catalytic PKA subunits in our screens; perhaps this is because *S. cerevisiae* has 3 paralogs (*TPK1/2/3*), and when only one is deleted, the other 2 may suffice. The gene encoding the regulatory subunit, *BCY1*, is essential and therefore was not tested.

In mitophagy, various MAPK pathways are positioned to phosphorylate Atg32p as their final target, allowing it to dimerize with Atg11p, initiating phagophore assembly. These kinases include the MAPKK Mkk2p, which activates Slt2p, both of which are also required in limS and discussed above in the MAPK cell wall stress cascade, and the MAPK Hog1p, required in limN and discussed in high osmolarity signaling. These 2 cascades have been shown to be necessary for mitophagy (Mao *et al*. 2011). Downstream of Hog1p is Ckb2p (YOR039W, limS), a regulatory subunit of casein kinase 2 (CK2). CK2 phosphorylates Atg32p in its regulation of mitophagy, particularly under nitrogen starvation conditions (Kanki *et al*. 2013). Here, we show its necessity under sulfur starvation. Although not present in any KEGG pathways, YLR407Wp (limN) has recently been shown to interact with CK2 (Michaelis *et al*. 2023). Several other genes encoding proteins functioning in autophagy (*ATG23*, YLR431C, limS and limN; *VAM7*, YGL212W, limS; and the following, which were all found in limited nitrogen: *ATG10*, YLL042C; *ATG2*, YNL242W; *ATG21*, YPL100W; *ATG29*, YPL166W; *KCS1*, YDR017C; *VPS33*, YLR396C; and *YSP3*, YOR003W) and mitophagy (*FMC1*, YIL098C, limS and limN; *ATG33*, YLR356W, limS; and *DNM1*, YLL001W, limN) were determined to be vital when nutrients were limited.

The redundancy of the signaling pathways around the main complexes speaks to their importance under starvation conditions. The major complexes were found here—TORC1, ATG1, membrane delivery proteins, Atg32p—but fewer of the peripheral proteins were. These “hubs” where incoming information is centralized are essential under our limited nutrient conditions.

### Role of endocytic pathways

There are many different reasons for which and ways in which the cell internalizes parts of its plasma membrane. Frequently, the cell does so in order to bring in nutrients or to recycle membrane components. KEGG separates some of these processes into endocytosis (4144) and phagocytosis (phagosome, 4145). While only ∼20% of the genes tested that KEGG lists in these pathways were found in our screens, the peptides encoded by them were often part of a multimeric complex, thereby increasing the ratio of functional proteins that were identified.

Motor proteins (4814) were also determined to be important when the cell was starved for sulfur or nitrogen. These proteins facilitate the movement of vesicles and large macromolecular structures. While motor proteins in general are crucial during other cellular processes such as nuclear divisions (4111 and 4113) and autophagies (4136 and 4139) discussed above, the specific motor proteins we identified in our screens are notated by KEGG to be involved in endocytic processes. In *S. cerevisiae*, 4 genes encode microtubular subunits—2 α, 1 β, and 1 γ. All are essential for growth under standard conditions aside from *TUB3* (YML124C, limS and limN), which encodes the α subunit responsible for only 10% of the cell's α-tubulin but whose inclusion results in more stable microtubules (Bode *et al*. 2003). In addition, the kinesins Smy1p (YKL079W, limS and limN) and Kip3p (YGL216W, limS) were revealed, both classified as N-KIFs, which move toward the positive end of their microtubule. Although genes encoding actin monomers and actin-associated proteins were not found here, it has been shown that Smy1p does not in fact interact with microtubules but rather is involved in myosin interactions (Hildebrandt and Hoyt 2000). The majority of the roles of these 2 proteins that have been conducted in yeast have been done with an eye toward cellular and nuclear division, and Kip3p has a crucial function during anaphase (Hildebrandt and Hoyt 2000).

In KEGG's endocytic pathway, phospholipase D, Spo14p (limS and limN), discussed in glycerophospholipid metabolism (564) above, works at the plasma membrane to generate phosphatidic acid. Although there is overlap in the literature between the mechanisms of endocytosis and sporulation in *S. cerevisiae*, researchers have found that the specifics are still muddy as to when and where each protein acts. While phospholipase D plays a major role in endocytosis in other species (Donaldson 2009), little has been done in yeast to elucidate the role of Spo14p in the process. The protein does, however, prove to be essential in sporulation, indicating a requirement of phosphatidic acid for that process (Rudge *et al*. 2004). This function, however, appears to be independent of End3p-mediated endocytosis, which is also required for sporulation (Morishita and Engebrecht 2005). Although not present in KEGG pathways, we found that End3p (YNL084C, limS) was crucial in our screen. PI(4,5)P_2_ is required for activation of Spo14p (Rudge *et al*. 2004)—we found 2 phosphatases that dephosphorylate PI(4,5)P_2_ (Inp52p and Inp53p), discussed above regarding IP and PIPs (562 and 4070), suggesting that a regulation of PI(4,5)P_2_ may be important for growth under starvation conditions. Ent5p (YDR153C, limN) contains an epsin-like domain and binds to clathrin and its adaptor proteins. As such, it is required for clathrin-mediated endocytosis (Duncan *et al*. 2003). Apl1p (YJR005W, limS) is the large subunit of the clathrin-associated protein complex (AP-2) in *S. cerevisiae*. These complexes require binding to PI(4,5)P_2_ in order to bind cargo receptors.

Found in the early/prevacuolar endosome, retromer acts to recycle receptors back to the plasma membrane. Here, we found one of the subunits, Vps29p (YHR012W, limN), and the associated nexin Snx3p/Grd19p (YOR357C, limS) (Hettema *et al*. 2003). Working to internalize the late endosomal membrane as the compartment matures into a multivesicular body, several peptides associated with endosomal sorting complexes required for transport (ESCRT)-III were identified. Vps24p (YKL041W, limS and limN) is an ESCRT-III subunit that binds to PI(3,5)P_2_, and the peptides Did2p (YKR035W-A, limS) and Vps60p (YDR486C, limS) are also ESCRT-III subunits but are not central. They function together with Ist1p (YNL265C, limS and limN) to mediate cargo sorting (Rue *et al*. 2008).

One of *S. cerevisiae*'s Rab5s, Ypt52p (YKR014C, limS and limN), is the monomeric GTPase associated with the early/prevacuolar endosome, allowing for docking of vesicles to their target membranes, so that v- and t-SNAREs may interact. Although not found in a KEGG pathway, the Rab5 guanine nucleotide exchange factor (GEF) Vps9p (YML097C, limS and limN) stimulates Ypt52p to release GDP, so that it may bind GTP and become active. These 2 proteins work together and with the other Rab5 paralogs to facilitate normal late-Golgi to endosome sorting (Nickerson *et al*. 2012) and vacuolar protein sorting with Vps33p (YLR396C, limN) (Peplowska *et al*. 2007). Roy1p (YMR258C, limS), an inhibitor of Ypt52p not in a KEGG pathway (Nickerson *et al*. 2012), was also found to be important when cells were undergoing starvation. Thus the tight regulation of this Rab may be important as the cell comes under stress.

KEGG does not present many Ras superfamily members in yeast as functioning in their pathways. A survey of KEGG's Brite database revealed 30 *S. cerevisiae* Ras superfamily genes, 24 of which were tested here. While we found 6 of those to be important when the cell was starved for nitrogen (2 of those also for sulfur), it is worth noting that of those 6, 3 are members of the Rho family (*RHO2*, YNL090W, limN; *RHO4*, YKR055W, limN; and *RHO5*, YNL180C, limS and limN), accounting for 60% of the Rho family tested (2 are essential). While Rho studies in yeast have focused on polarized growth and oxidant-induced cell death (Gong *et al*. 2013; Singh *et al*. 2019), Rho members of other species are involved in not only actin polymerization but also microtubule stabilization (Mosaddeghzadeh and Ahmadian 2021). Finally, although KEGG lists it in endocytosis, the ADP ribosylation factor (ARF)-activating GEF Gea1p (YJR031C, limS) appears to have a role in the maintenance of the Golgi rather than in endocytosis (Peyroche *et al*. 2001); Arfs generally are known to be involved in coat protein complex I (COPI) or clathrin coat recruitment at the Golgi.

KEGG separates the phagosome pathways (4145) into 2 processes: conventional and ER-mediated phagocytosis. Aside from 2 subunits of Sec61 (Ssh1p and Sbh1p, both limS and protein export (3060) above), the 2 other ER-mediated pathway genes were not discovered in our screens. However, peptides involved in the conventional phagocytosis, related to the early phagosome and the phagolysosome particularly, were discovered. Ypt52p, yeast's Rab5, is also active in phagocytosis as well as endocytosis described above. In addition, Mrl1p (YPR079W, limS) is similar to mannose-6-phosphate receptors and is likely involved in sorting and delivering proteins to the vacuole (Whyte and Munro 2001). These 2 proteins, as well as the phagocytic vesicles, associate with microtubules as they direct vesicular movement. Here again we see the importance of microtubules, with Tub3p playing a role in phagocytosis as well as endocytosis. Finally, although Vma5p (YKL080W, limS), subunit C of the vacuolar ATPase involved in decreasing the pH in the early phagosome, was found here, the other 9 subunits tested were not statistically relevant.

### Transcription factors

To extend our analysis of identified genes, we queried our limS and limN genes for those previously determined to be transcription factors. Previously, Gordân *et al*. (2011) analyzed transcription factors from several studies, investigating 190 transcription factors from *S. cerevisiae*. Of those, 168 were represented in the knockout library, and 39 were identified in our screen. Several of those identified have been implicated in regulating some of the processes discussed above, with 6 identified in our KEGG analysis. Because the KEGG pathways for MAPK signaling, cell cycle, and meiosis (4011, 4111, and 4113, respectively) are specific to *S. cerevisiae*, these species-specific transcription factors were included in the database. Six genes, all of which were identified by poor growth under limited nitrogen conditions, were found in KEGG: *DIG1*, *RLM1*, *MBP1*, *SKO1*, and *SWI6* have been discussed above. The deletion of the latter 2 weas also found to cause poor growth in limited sulfur medium. Pho4p (YFR034C, limS and limN) may compete with Cbf1p (not found), which is known to bind upstream of genes involved in methionine metabolism (Zhou and O'Shea 2011). Pho4p binds to motifs found upstream of at least 80 genes, but most are not regulated as a response to phosphorous starvation. *PHO84* (limN), is one of its more commonly studied targets and is involved in PI signaling. While Pho4p is annotated in KEGG's yeast cell cycle pathway, no proteins adjacent to it in KEGG were identified in our screens.

A number of transcription factors associated with regulation of genes encoding transporters were revealed. Cup9p (YPL177C, limS and limN) is a transcription repressor that negatively affects expression of at least 2 peptide transporters, *PTR2* and *OPT2*, neither of which were found here, as well as *SIR2* (YDL042C, limN), a histone deacetylase which Cup9p represses under heat stress (Hauser *et al*. 2001; Laskar *et al*. 2015). Independently, the SPS amino acid–sensing pathway (Ssy1p, Ptr3p, Ssy5p) positively regulates *PTR2* and *OPT1* (YJL212C, limN), an oligopeptide transporter and homolog of *OPT2*, with Ptr2p expression increased in poor nitrogen sources and the expression of Opt1p increased in limited sulfur (Wiles *et al*. 2006). Unfortunately, none of the 3 members of SPS were present in the library and therefore could not be tested, particularly since they are connected with Tor1p, discussed above, especially since Tor1p responds to the presence of amino acids (González and Hall 2017). We were not, however, challenging cells to survive on sources of amino acids as sulfur or nitrogen supplies. In addition, both Stp1 (YDR463W, limN) and Stp2 (YHR006W, limS) paralogs participate in the expression of *BAP3* (not found), an amino acid transporter, and act through the SPS pathway (de Boer *et al*. 2000; Omnus and Ljungdahl 2014). Omnus and Ljungdahl have also shown that deletion of *ASI1*, *ASI3* (paralogs, neither found), and *ASI2* (YNL159C, limS) disrupt the need for the SPS pathway, constitutively activating Stp1p and Stp2p gene targets.

Pdr8p (YLR266C, limS) and its paralog Yrr1p (YOR162C, limN) activate genes acting in multidrug resistance, several of which are transporters (Hikkel *et al*. 2003). We also identified several targets of these transcription factors, namely *IMA5* (YJL216C, limN), encoding α-glucosidase; *AZR1* (YGR224W, limS and limN), encoding a plasma membrane drug resistance transporter; *YLR046C* (limS and limN), uncharacterized; *LAF1* (YMR102C, limN), involved in transportation of ergosterol from the plasma membrane to the ER; *PBL1* (YMR006C, limS), phospholipase B; *PDR16* (YNL231C, limN), a PI transfer protein; and *YPR127W* (limS and limN), a pyridoxine (vitamin B6) dehydrogenase (Lucau-Danila *et al*. 2003). Other genes involved in multidrug resistance were also identified: *PDR10* (YOR328W, limS), a transporter, and both *PDR16* (YNL231C, limN) and *PDR17* (YNL264C, limN), PI transfer proteins.

Heme-activated transcription factors were identified here. Hap2p/3p/4p/5p is a CCAAT-binding factor that likely regulates most nuclear genes involved in respiration (Mao and Chen 2019). Hap2p (YGL237C, limN), Hap3p (not found), and Hap5p (YOR358W, limS) form a trimer to which Hap4p (YKL109W, limS) is recruited, bringing the activation domain to the complex. It has also been determined that a similar complex, Hap2p/3p/5p/Gln3p, regulates some genes involved in nitrogen metabolism when cells are grown on a poor nitrogen source, such as proline (Hernández *et al*. 2011). It is interesting to note that we identified neither Hap3p nor Gln3p in either screen, nor did we identify the known targets of this complex, namely *GDH1* and *ASN1*, nor their respective paralogs, *GDH3* and *ASN2*. The difference could be in the nitrogen source provided (none beyond minimal requirements of amino acids and vitamins vs an abundance of proline) or the possibility that when one gene was knocked out, its paralog was sufficient for growth in limS and limN media. Hap4p, however, does not seem to be required for the activation of these genes under poor nitrogen conditions, but we identified its possible requirement here when sulfur was limited, suggesting a role for the complex in a broader area of nutrient response than only respiration and nitrogen genes.

Another heme-activated transcription factor, Rox1p (YPR065W, limS), was observed to be important during sulfur starvation, along with *ECM22* (YLR228C, limS). These both encode transcription factors that work downstream of Hog1p (limN) and Sko1p (limS), discussed above in response to high osmolarity, to regulate the expression of *ERG* genes, needed for ergosterol synthesis (Montañés *et al*. 2011). Rox1p negatively affects Ecm22p and *ERG* expression, while Ecm22p upregulates them. Ecm22p, however, also positively regulates filamentous growth, which can result under starvation conditions (Woods and Höfken 2016).

Several other transcription factors are of note, in that they relate to processes found throughout our KEGG analysis. Vhr1p (YIL056W, limN) is required for expression of *BIO5* (limS), addressed above with vitamin B7, biotin (Weider *et al*. 2006). Little work has been done to investigate further targets of Vhr1p, but given its importance in activation of *BIO2* and *BIO5*, its potential relation to expression of other genes found in this study, *BIO3* (limS and limN) and *BIO4* (limS) may be relevant.

In wine strains, Walkey *et al*. (2012) have shown that the transcription factor Tda9p (YML081W, limS and limN) is required for expression of the gene encoding the major mALD, *ALD4* (limS), which we discussed above as being annotated to function in 6 amino acid and 3 carbon metabolic pathways in KEGG.

Yap1p (YML007W, limS and limN) and Skn7p (YHR206W, limN) are responsible for gene regulation under a variety of oxidative stresses (Brombacher *et al*. 2006). Of the 4 genes, Brombacher *et al*. confirmed were positively regulated by both transcription factors; *TSA1* (YML028W, limN), a gene encoding a peroxiredoxin with chaperone activity (MacDiarmid *et al*. 2013), was also found to be required here for growth in limited nitrogen. While it is unclear as to the role Yap1p and Skn7p play in expression of other oxidative stress response genes, we did identify a number of genes that have been implicated in this stress response in other studies, discussed above.

Ino2p (YDR123C, limN) heterodimerizes with Ino4p (not found) to repress genes involved in phospholipid synthesis (Jesch *et al*. 2005). Three known targets of Ino2p, *CKI1* (limN), *MHO1* (YJR008W, limS), and *INO2* itself, were also required here. We also identified 26 more genes previously described by Jesch *et al*. as being differentially regulated under inositol conditions, including the previously discussed *PHO84*, *BIO5*, *OPT1*, and *GCV2* (Supplementary Table 2).

Finally, Met28p (YIR017C, limS) has been shown to be required for the transcription of several genes involved in the sulfate assimilation pathway (KEGG 920), which we did not observe. Perhaps, this is because there were not sulfur salts available for the cell to make use of. There are few studies on the targets of Met28p—it may therefore have wider targets than simply sulfate assimilation.

### Gene Ontology analysis

Of course, few genes encoding proteins are transcription factors, and we found that only 39 genes are identified in at least one of our screens, or 3.3% [only 4.1% of the tested genes are transcription factors annotated by Gordân *et al*. (2011)], with 27 required for growth in limS and 23 required in limN. When combined with our KEGG analysis of 337 genes, only 369 genes were systematically analyzed. While there are surely proteins missing annotations in the KEGG pathways database, not all proteins have a function that will place them into one of the currently maintained KEGG pathways; however, every protein will have at least one each of a biological process, molecular function, and cellular component. In seeking to extend the systematic analysis of these gene sets, we queried the PANTHER GO-Slim databases (Thomas *et al*. 2022) for these 3 categories and increased the systematic coverage of our identified genes by 360 genes, bringing the coverage to 729 of our 1,180. All of these additional genes are either verified or uncharacterized at SGD. Of the 451 genes we were unable to systematically analyze, 361 are annotated as verified or uncharacterized by SGD, while the remaining 90 are annotated as dubious ORFs or have another annotation at SGD.

All PANTHER GO-Slim categories that were overrepresented in at least one dataset (limS, limN, or the intersection of limS and limN) were determined by comparing the respective gene lists with the list of genes tested with the knockout library using the PANTHER Overrepresentation Test (Mi *et al*. 2019). A set of overrepresented categories found in the library when compared with the list of 6,060 *S. cerevisiae* genes housed at PANTHER was also retrieved. While there were several GO-Slim categories found to be statistically overrepresented in the list of library genes, no GO terms were found to be significant in the other lists when compared with the tested genes.

Of the ∼3,000 GO-Slim terms, 1,182 are overrepresented in at least one of our datasets. Of these terms, 689 are a biological process, 296 a molecular function, and 197 are a cellular component. For 20–25% of the terms found in each category, only 3 or fewer genes from those 4,934 we tested are annotated. With so few genes annotated with each term, GO terms for which we found all annotated genes in our screens did not provide a statistically relevant *P*-value, even before applying a correction for multiple testing (Supplementary Table 4). We still found trends in overrepresented useful in understanding our datasets. We focused on those GO-Slim categories with at least 15 genes represented in the knockout library that were also overrepresented at least 2-fold in limS, in limN, or in the intersection between limS and limN. This subset of GO-Slim terms contained 37 biological process, 12 molecular function, and 15 cellular component terms (Table 1). Many of these categories were related to phospholipids, phosphate metabolism, and cellular trafficking, correlating to our findings from KEGG.

**Table 1.** Go-Slim terms identified via PANTHER with 15 or more genes tested in the knockout screens found to have at least 2.0×-fold enrichment in genes found to be required for growth in limited sulfur medium (limS), in limited nitrogen medium (limN), or in both (limS and limN).

GO category	GO terms
limS medium	
Biological process	Fatty acid metabolism, monocarboxylic acid biosynthesis
Molecular function	—
Cellular component	Cell tip, cell pole, RNA polymerase II transcription regulator complex
limN medium	
Biological process	Amino acid catabolism, glycerophospholipid biosynthesis
Molecular function	GTPase, endopeptidase, hydrolase, C-N linear amides
Cellular component	Ubiquitin ligase complex, organellar ribosome, mitochondrial ribosome
limS and limN (intersection of both screens)	
Biological process	Reproduction, lipid metabolism, fatty acid metabolism, membrane lipid metabolism, phospholipid metabolism, sphingolipid metabolism, lipid transport, intracellular iron ion homeostasis, endocytosis, vesicle fusion, lipid biosynthesis, phospholipid biosynthesis, nucleoside triphosphate biosynthesis, purine nucleoside triphosphate biosynthesis, ribonucleoside triphosphate metabolism, ribonucleoside triphosphate biosynthesis, purine ribonucleoside triphosphate metabolism, response to abiotic stimulus, lipid localization, lipid catabolism, regulator of phosphate metabolism, sexual reproduction, reproductive, regulator of protein modification, cellular lipid catabolism, cellular lipid metabolism, glycerolipid biosynthesis, organophosphate catabolism, membrane lipid biosynthesis, glycerophospholipid biosynthesis, regulator of cellular component organization, regulator of phosphorus metabolism, membrane fusion, monocarboxylic acid biosynthesis, import into cell, meiotic cell cycle
Molecular function	Monoatomic cation channel, channel, lipase, hydrolase, C-N linear amides, carbohydrate kinase, phosphatase regulator, protein phosphatase regulator, passive transmembrane transport, metal ion transmembrane transport, transition metal ion binding
Cellular component	Ubiquitin ligase complex, storage vacuole, lytic vacuole, fungal-type vacuole, fungal-type vacuole membrane, vacuolar membrane, cell cortex, cell tip, cell pole, lytic vacuole membrane, supramolecular polymer, supramolecular fiber

### Verification

After querying KEGG, PANTHER, and previous studies, we chose 184 strains that showed reduced growth in our no-sulfur or limited nitrogen media to verify through additional assays. We selected a range of deletants focusing on those that were identified in both screens, but also choosing some that were only found in one. We ensured that there were a mix of genes represented that were found in interesting KEGG pathways and transcription factors and other genes not annotated in KEGG. Finally, we chose a selection of genes not only annotated as being valid genes in SGD but also some that were uncharacterized and even dubious.

**Fig. 3.**
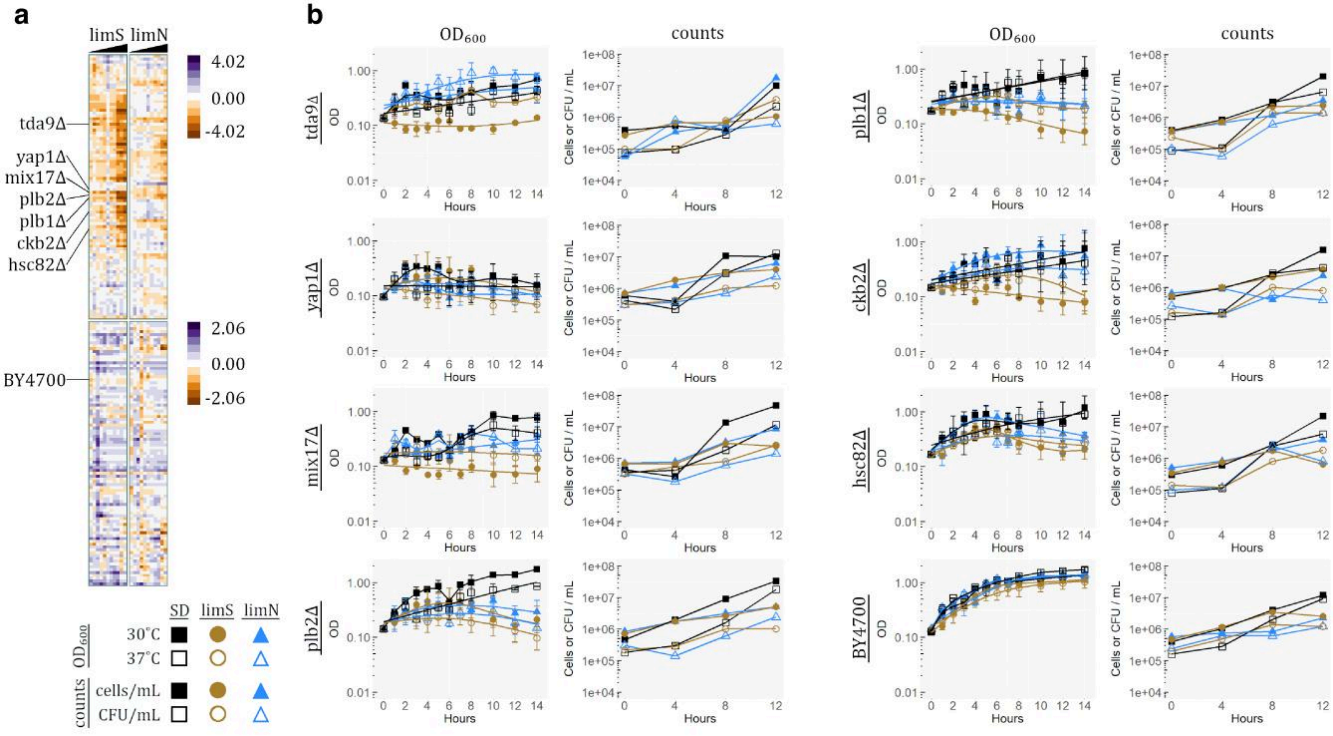
Verification of 184 deletants identified to be required for growth in limS or limN when compared with their growth in SD. a) Heat maps showing M-values of growth at 30°C for which those with less growth in limited medium compared with SD are negative (orange) and those with more growth in limited medium are positive (purple) from 1 to 14 h, left to right. Assays were conducted in two 96-well plates (top and bottom), and deletants are arranged in plate order. b) Growth curves for 7 deletants and control (BY4700) in SD (black squares), limS (yellow circles), and limN (blue triangles). OD_600_ values plotted are taken from the 96-well plate verification assays at 30°C (closed shapes) and 37°C (open shapes). Cell and CFU values were obtained from growth assays conducted in 50 mL cultures, with hemocytometer cell counts and aliquots for CFUs taken from the same cultures at the same times. Petri dish images from which CFUs were determined are available in Supplementary Fig. 4.

Growth assays were conducted in a manner similar to those of the original screen, but optical densities were determined every hour from 0 to 8 h and then every 2 h until 14 h was reached. Cells were grown at both 30 and 37°C. All verification was completed on the same day, and each strain was normalized between plates by determining the average of all 12 wells at time 0 to account for variations in inoculation (3 media in each of 2 temperatures, in duplicate). *M*-values were then determined for each set of comparisons to be made (Supplementary Table 5), such that negative values indicate poorer growth in limited medium compared with in SD, as well as poorer growth at 37 when compared with that in 30°C compared with a control strain. In general, deletant strains were verified to grow worse in limS over time, particularly in the later hours of the assay (Fig. 3). Deletant strains also grew worse in limN, but the effect was not as strong. While deletants showed a marked difference in growth between 30 and 37°C, again, particularly at the later hours, there was less difference between growth at the 2 temperatures when cells were challenged to grow in limited media (Supplementary Fig. 3).

To determine if some of the effects observed were due to loss of viability as opposed to slower or reduced growth, we further examined a random selection of 7 deletion strains to determine the cell concentration by counting rather than relying solely upon OD. Cultures were grown at 30°C for 12 h, with aliquots taken every 4 h to both determine cell concentration as well as to determine viability via a spot assay (Fig. 3; Supplementary Fig. 4). Proteins encoded by those genes deleted in these strains that were discussed above were Plb1p (limS) and Plb2p (limS and limN), both phospholipase Bs but not paralogs; Ckb2p (limS), a regulatory subunit of CK2 involved in mitophagy; Tda9p (limS and limN), a transcription factor required for *ALD4* expression; and Yap1p (limS and limN), a transcription factor involved in oxidative stress. Two additional strains were observed, the products of genes deleted in which were not discussed above. These were Mix17p/Mic17p (YMR002W, limS and limN) and Hsc82p (YMR186W, limS and limN). *MIX17* encodes a protein localized to the intermembrane space of the mitochondrion, and is a twin CX_9_C protein acted on by Mia40p (Gabriel *et al*. 2007). Unlike other genes in its protein class, it does not appear to be important in respirative growth on nonfermentable carbon sources but like most of its class, its deletion does have a respiratory deficiency (Longen *et al*. 2009). Hsc82p is found in the KEGG pathway Protein Processing in the ER (4141). Its paralog, Hsp82, was not found, and these paralogs are the only Hsp90s in *S. cerevisiae*. In *Sc. pombe*, this family is required for maintenance of CDK kinases Wee1p and Mik1p (Goes and Martin 2001).Colony forming units (CFUs) paralleled cell counts over time, suggesting that these deletants challenged to grow in limS or limN media did not lose viability but rather slowed growth, some perhaps approaching stationary phase.

### Conclusion

Here, we investigated genes that have a role in maintaining culture growth early in the growth of cells in nutrient-limiting conditions of no source of sulfur (limS) or no readily accessible source of nitrogen (limN). Under these conditions, cells were required to use the available sulfate and ammonium internally carried from overnight medium, and their stores of these biologically available elements contained within other metabolites. While wild-type cells, and indeed the majority of deletants, showed no different culture growth phenotype when challenged with starvation for 4 h, we found 1,180 genes that, when deleted, could not sustain growth when cells were starving when compared with the deletant's growth in the complete, SD medium; 732 were required when sulfur was absent, while 761 were required when nitrogen was severely limited. The overlapping 313 genes, nearly 3 times as many as expected, potentially suggest a characterization of a more general starvation response. Akin to this, Gasch *et al*. (2000) first described the environmental stress response (ESR) transcription profile, sets of genes that were transcriptionally regulated in response to a multitude of stressful conditions. Genes we found to be required for culture growth in these starvation conditions overlap with those identified by Gasch *et al.* as transcriptionally responding to stress, as well as with those deletants determined by Auesukaree *et al*. (2009) to be required for growth under various stress. Our data do not share significant overlap with these datasets (Supplementary Table 2). Gasch *et al.*, however, measured transcriptional response rather than growth response, while Auesukaree *et al.* was not examining nutrient starvation. Further work in this area may elucidate an understanding of genes not only required for growth when cells are deprived of a specific nutrient but also those that more generally respond to various starvations.

While we did not seek for our laboratory growth conditions to mimic that of wild conditions, the limited nutrient media are perhaps better aligned with what wild yeast would be more likely to encounter, in that in the wild there is not an abundance of ammonium and sulfate salts. The genes deleted in the knockout libraries have been deemed nonessential because they could be removed and culture growth still maintained in synthetic complete laboratory conditions. Here, we see that when cells do not have an abundance of these available elements, these genes are essential. Throughout the 2 screens, we often found only one paralog to be of importance and not the other. Evangelisti and Conant (2010) noted the same when analyzing ribosomal protein gene representation in the knockout libraries. Occasionally, we found one paralog to be of importance in limited sulfur while the other was important in limited nitrogen. This paralog effect therefore likely extends to other processes and growth conditions; while paralogs may seem to have the same function, they cannot necessarily substitute for each other. Even with these differences in paralogs identified, we observed a greater than expected number of genes that overlap between our 2 screens.

In analyzing the 1,180 for placement of their products in metabolic and signaling pathways, we identified 337 present in the KEGG pathways database, finding pathways that were affected to be wide-ranging, including the metabolisms of all macromolecules and some vitamins, and cellular processes and signaling such as PI signaling, vesicular fusion, autophagy, and endocytosis. We found that in metabolism, several amino acid metabolic pathways were affected, particularly that of cysteine and the adjacent glutathione metabolism. Also of note were 3 proteins that work in a multitude of amino acid pathways: Ald3p and Ald4p, 2 ALDs were also involved in carbon metabolism, and Aro8p, a transaminase was also involved in ubiquinone biosynthesis.

Saldanha *et al*. (2004) found that when media were limited in “natural” nutrients, that is to say phosphate or sulfur as opposed to being limited in leucine or uracil as needed to fulfill an auxotrophy, cells demonstrated a stress response in transcription concurrently with a coordinated cell cycle arrest just as those natural nutrients were completely depleted. They conclude that before the arrest, cells are “poor, not starving” and that their growth rates are modulated to account for the reduced nutrients present. Here, therefore, we have observed either those deletant strains that must slow their growth rate sooner due to their gene deficiency because either they cannot access or utilize the paucity of nutrient stores available within them or those strains that have failed to slow their rate, thereby using up nutrients sooner due to dysregulation of their growth rate.

Although it may not be readily apparent why the central metabolism and fatty acid and glycerolipid synthetic pathways may be important when the cell has a scarcity of sulfur or nitrogen, it strikes us that 2 possible effects may be at play. Above, we suggest that perhaps the stress from a dearth of biologically available nitrogen or sulfur, even though carbon is in abundance, may be too much for the cell to overcome when combined with a gene deletion affecting carbon utilization, thereby presenting the phenotype of reduced growth in either or both limiting media. Perhaps, the central metabolism genes we identified were not essential enough to prevent them from being knocked out, but the added and unrelated stress of a different absent nutrient passed the deletant's tipping point.

However, Boer *et al*. (2008) have observed that cells starved for sulfur or phosphorus utilize their carbon source at a slower rate when compared with those undergoing auxotrophy starvation, which speed through carbon in a manner they refer to as “glucose wasting.” Cells challenged to grow in these “natural” limiting conditions must therefore be regulating this slower growth by slowing their carbon utilization. In starving deletant cells of available nitrogen or sulfur, we have thus identified not only genes required to relay starvation signals to cellular components in order to elicit the proper response (e.g. autophagy) and those whose proteins directly metabolize alternative sources (e.g. cysteine) but also genes whose products regulate carbon usage. Boer *et al.* proposed that Tor1p is critical in relaying the signal to reduce glucose usage—indeed, we did find that *TOR1* was required for normal cell growth during nitrogen starvation, although not sulfur. Perhaps, those “nonessential” central metabolism genes we identified are essential after all for adaptation and cellular responses to “natural” nutrient stress.

In addition, we note that specific lipids are important in the structure, function, and location of integral membrane proteins. The metabolism of 2 particular lipids stands out from our data: cardiolipin and PIs. Cardiolipin is known to interact with and be required for proper functioning of all ETC components, ADP/ATP carriers between the cytosol and mitochondria, and mitochondrial ATP-synthase (Paradies *et al*. 2019), directly tying the effects of starvation that we see in lipid metabolic pathways to oxidative phosphorylation. The 4 fatty acid tails of cardiolipin are thought to play a pivotal role in establishing the curvature of the mitochondrial cristae, along with supporting the oligomerization of ATP-synthase, which also establishes and requires the cristae for function (Acehan *et al*. 2011). Relatedly, we found members of all complexes of the mitochondrial ETC aside for the single yeast NADH-ubiquinone reductase gene, which is essential. Specifically, it has been shown that the ETC of *S. cerevisiae* plays a role in the regulation of glucose starvation response (Lewis *et al*. 2021). With the fact that glucose usage is regulated during times of natural starvation, it can be unsurprising that we have also found central metabolism here.

Further, we have unsurprisingly found components of autophagy and mitophagy in both of our screens. As cells are faced with a dwindling supply of available sulfur or nitrogen, they must increase the recycling of molecules in order to build new components in response to the changing environment. Although these are largely separate processes in yeast, we found those proteins that are present in both, namely Atg11p and Slt2p. We also identified the main hub in each process: Atg13p in autophagy and Atg32p in mitophagy. Proteins acting in mitophagy that we found to be crucial during nutrient starvation were tightly grouped, converging on Atg32p. With ∼1,000 proteins acting in the yeast mitochondrion (Malina *et al*. 2018), it is clear that this organelle needs to be tightly regulated in the face of various nutrient starvations, not only carbon starvation.

The other lipid, or rather family of lipids, that our data highlight is PI and its PIPs. Genes involved in PI metabolism are not generally discussed among those found upregulated in nutrient shortages. PIPs and their metabolites effectively label cellular compartments and are in essence read by proteins involved in delivering cargo to the correct compartment. Relatedly, we have found that genes encoding proteins acting in endocytic pathways are of importance during sulfur- and nitrogen-limiting conditions, and these pathways have not readily been identified before as important in starvation response. Within the varieties of endocytosis, we particularly note SNARES, docking proteins, and proteins involved at the plasma membrane, in the early endosome, and at the trans-Golgi network. We also found a substantial number of proteins involved in vesicular movement, such as Rabs, Arfs, Rhos, and their regulators. Many of the proteins involved in these types of intracellular transport find their targets by coincidence detection of a particular PIP found in the membrane of the target compartment.

In their review, Broach (2012) describes a host of genes whose transcriptional regulation is affected by nitrogen starvation. The proteins are encoded by these gene functions in the vacuole, late endosome, and Golgi. While this aligns somewhat with our findings, it is at odds with others. We did not observe many late endosomal, lysosomal, or vacuolar proteins to be crucial in starvation conditions. The difference may lie in the difference in screen—examining transcription profiles vs the effect of gene deletion. In addition, throughout the signaling pathways we identified, microtubules and their associated proteins appeared to be crucial to cell culture growth. Microtubules have recently been identified as the main cytoskeletal protein responsible for mitochondrial localization (Fernández Casafuz *et al*. 2023), and their importance in vesicular movement has been understood. Genes involved in microtubule processes have been discovered to be of importance during phosphate starvation (Boer *et al*. 2008), but in general, microtubule regulation is also not a process commonly discussed in nutrient insufficiency responses. The disruption of the genes encoding the machinery in these processes speaks to the importance of endocytotic, autophagic, and related intracellular trafficking pathways in response to nutrient starvation. We also noted that while supplying cells with a variety of vitamins, biosynthetic pathways of a few of them contained a large proportion of proteins required during starvation. These particular vitamins and vitamers, primarily B3 and B6, were cofactors or substrates of other proteins revealed in our screens. In fact, over 30% of the proteins that require pyridoxine were crucial to survival in the absence of sulfur or ready source of nitrogen. The necessity of these coenzymes is not often uncovered in transcriptional analyses of starvation response.

Broach (2012) notes that rather than gene expression changes directly impacting changes in metabolite concentrations, regulation at the protein level by allosteric effectors and posttranslational modification is more likely to affect metabolism in yeast. Our data add to the understanding of these processes by assaying genes whose function is required, not just examining those genes whose mRNA levels are modulated. Even so, more work needs to be conducted in this area to get a full account of the cell's response to nutrient starvation.

## Supplementary Material

jkaf074_Supplementary_Data

## Data Availability

All data are provided in Supplementary Tables 1–5. The authors affirm that all data necessary for confirming the conclusions of the article are present within the article, figures, and Supplementary Material. Supplemental material available at G3 online.
