# Supplementary material for: A Saccharomyces cerevisiae knockout screen for genes critical for growth under sulfur- and nitrogen-limited conditions reveals intracellular sorting via vesicular transport systems
Source: G3 (Bethesda). 2025 Apr 10;15(7):jkaf074. doi: 10.1093/g3journal/jkaf074 (PMC12239629; doi:10.1093/g3journal/jkaf074)
Supplement: jkaf074_Supplementary_Data [file jkaf074_supplementary_data.zip › Supplemental_Material_Legends_G3-2024-405460.pdf]

Supplemental Material

Supplemental Table S1: Full data set of screens, including normalized data of each replicate for SD, limS, and limN, their p-values, and their coefficients.

Supplemental Table S2: Genes found to be statistically relevant in at least one screen, including their p-values, coefficients, and KEGG pathway IDs if annotated. Notations are included when genes found in previously published screens. Red text indicates p-values <0.05.

Supplemental Table S3: All KEGG pathways in which at least one deletant was found to have statistically less growth in at least one limiting medium as compared to growth in SD. Included are totals of genes/proteins and reactions/complexes present in yeast, in the screen, and found in each medium.

Supplemental Table S4: All PANTHER GO-Slim terms for which at least one deletant was found to have statistically less growth in at least one limiting medium as compared to growth in SD which were overrepresented in at least one data set. Included are totals of GO terms annotated in yeast, in the screen, found in each medium, and found in the intersection between both screens, along with the expected number and fold enrichment.

Supplemental Table S5: Full data set from verification experiments, including data normalized to hour 0 of each replicate for SD, limS, and limN at 30 °C and 37 °C, and their M-values.

Supplemental Figure S1: KEGG pathway 04138 Autophagy - Yeast. Colors represent at least one protein annotated at the node/reaction had statistical relevance in limS only (yellow), in limN only (blue), or in both limS and limN (green). Those nodes that were tested but no proteins were found to be relevant are grey, and those nodes present in *S. cerevisiae* but were untested are black. Nodes in white are not annotated in KEGG to exist in *S. cerevisiae*.

Supplemental Figure S2: KEGG pathway 04139 Mitophagy - Yeast. Colors represent at least one protein annotated at the node/reaction had statistical relevance in limS only (yellow), in limN only (blue), or in both limS and limN (green). Those nodes that were tested but no proteins were found to be relevant are grey, and those nodes present in *S. cerevisiae* but were untested are black. Nodes in white are not annotated in KEGG to exist in *S. cerevisiae*.

Supplemental Figure S3: Heat maps of verification M-values comparing growth over time in a limiting medium compared to that strain's growth in SD and M-values comparing growth at 37 °C to growth at 30 °C for each medium.

Supplemental Figure S4: Yeast spot assays. Cells were grown in limS, limN, and SD for 12 hours, and cell suspensions were removed every four hours, serially diluted, and 5 μL spotted onto YEPD plates.
