# Supplementary figures and images for: A Saccharomyces cerevisiae knockout screen for genes critical for growth under sulfur- and nitrogen-limited conditions reveals intracellular sorting via vesicular transport systems
Source: G3 (Bethesda). 2025 Apr 10;15(7):jkaf074. doi: 10.1093/g3journal/jkaf074 (PMC12239629; doi:10.1093/g3journal/jkaf074)

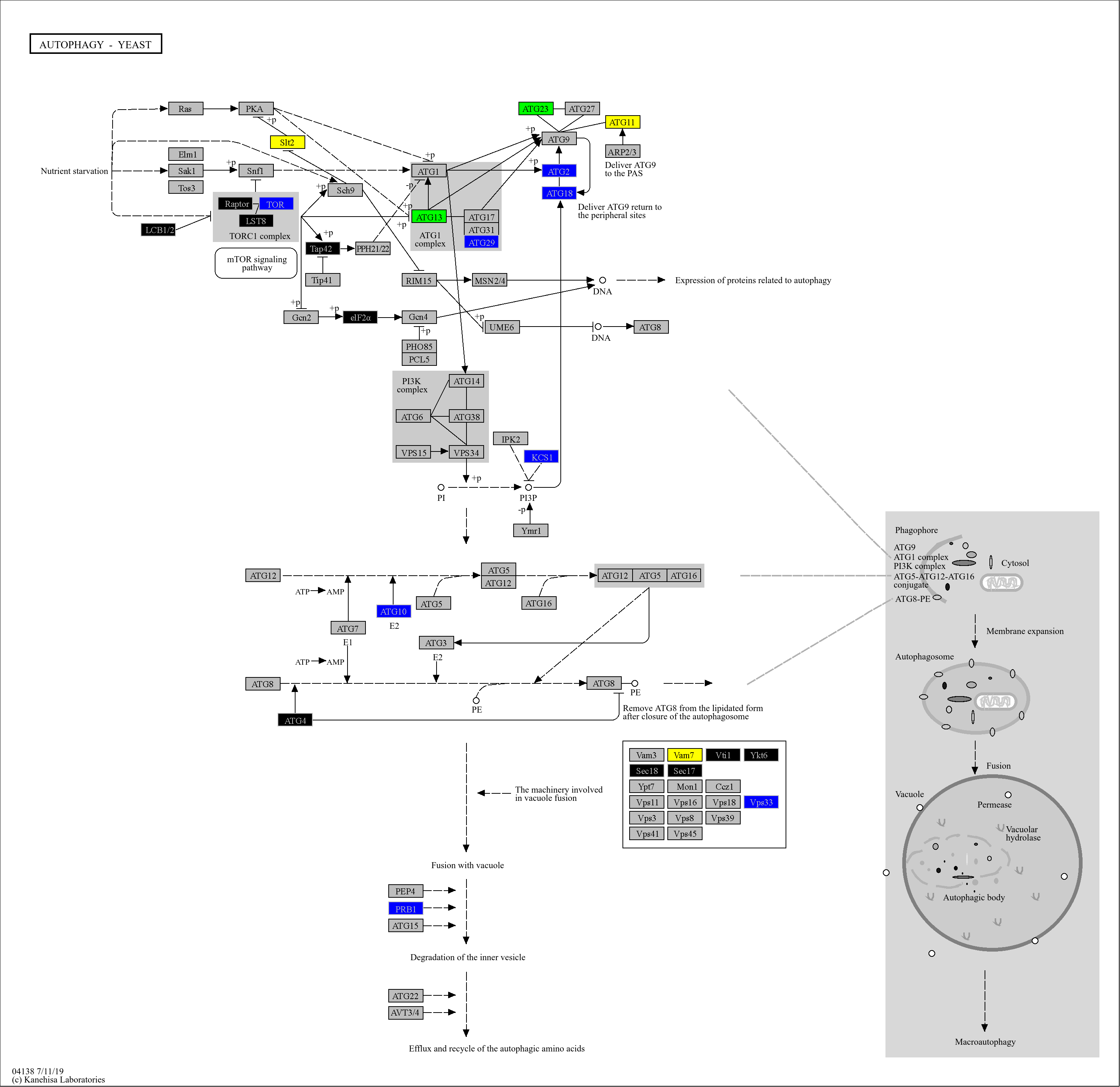

Supplement: jkaf074_Supplementary_Data [file jkaf074_supplementary_data.zip › Supplemental_Figure_S1_G3-2024-405460.png]

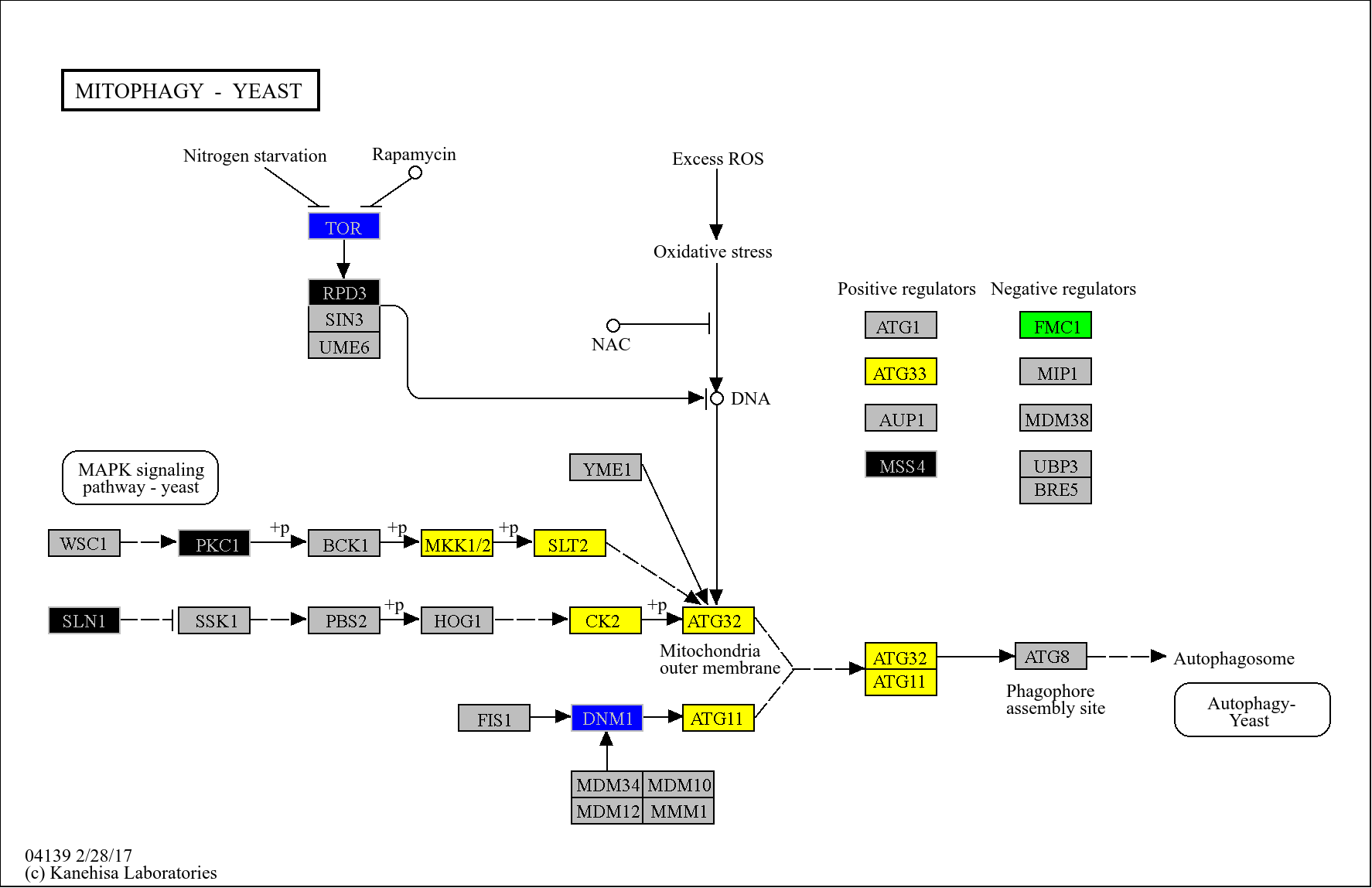

Supplement: jkaf074_Supplementary_Data [file jkaf074_supplementary_data.zip › Supplemental_Figure_S2_G3-2024-405460.png]

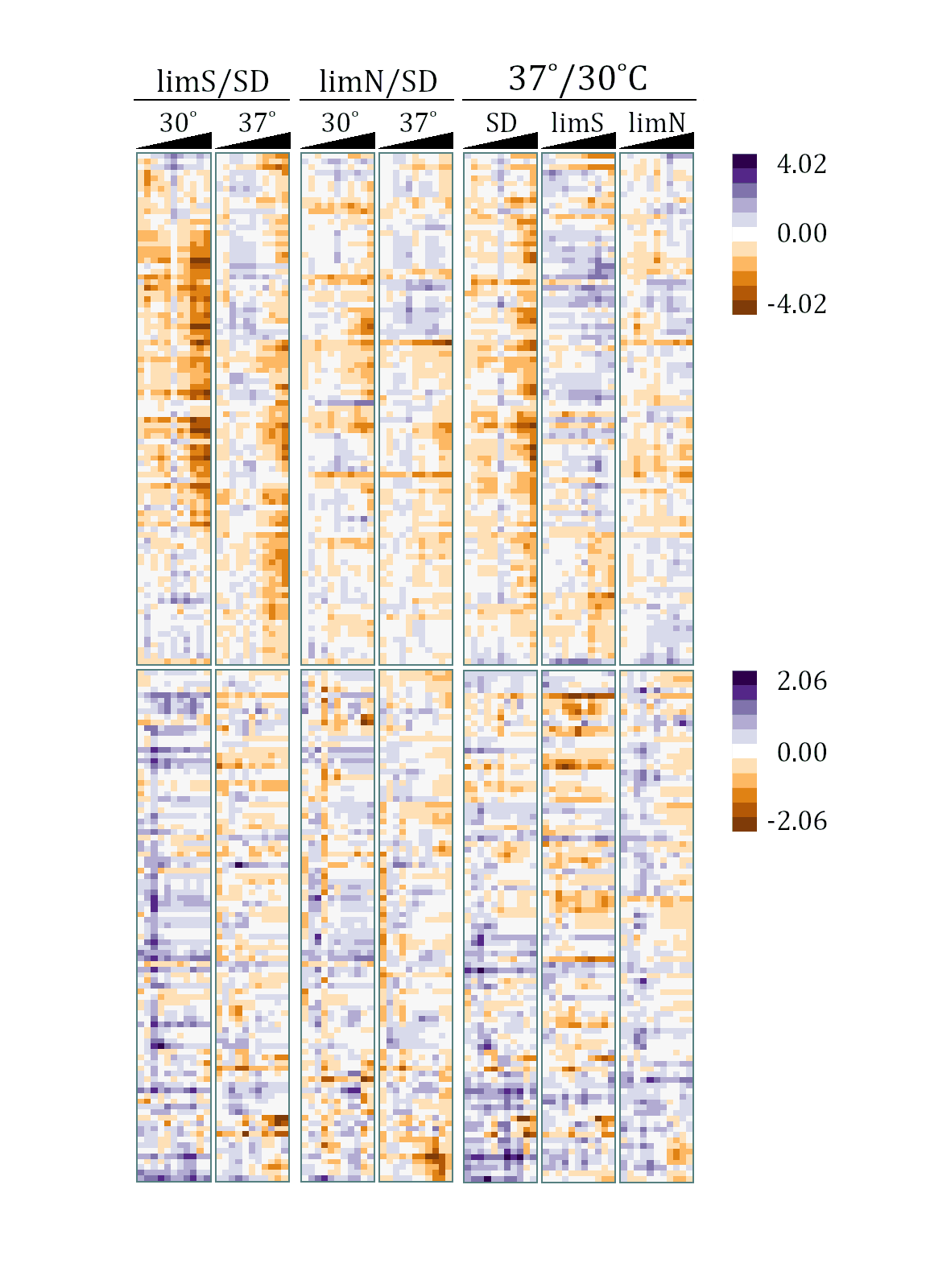

Supplement: jkaf074_Supplementary_Data [file jkaf074_supplementary_data.zip › Supplemental_Figure_S3_G3-2024-405460.png]

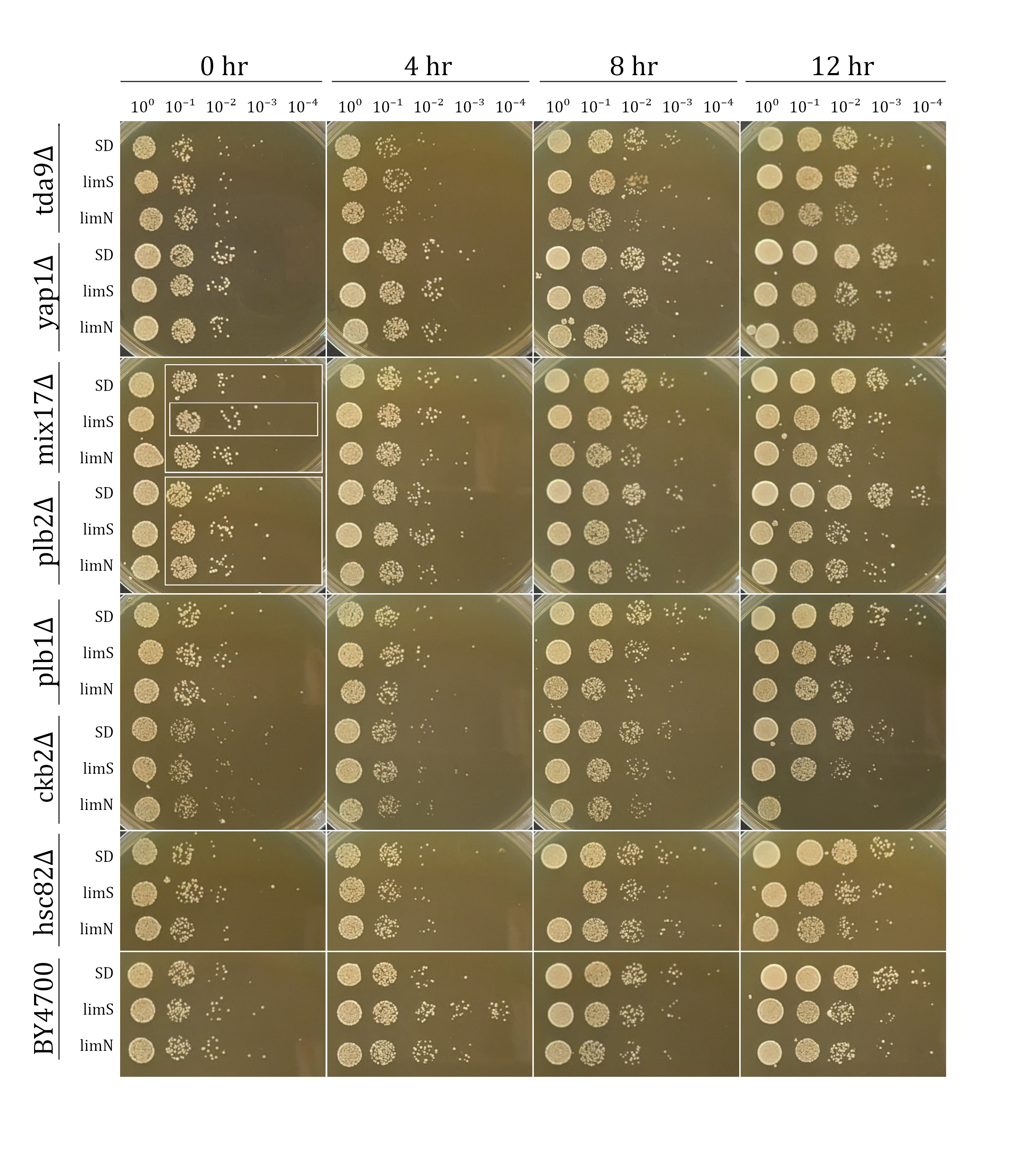

Supplement: jkaf074_Supplementary_Data [file jkaf074_supplementary_data.zip › Supplemental_Figure_S4_G3-2024-405460.png]
